# Multiple sclerosis risk variants alter expression of co-stimulatory genes in B cells

**DOI:** 10.1093/brain/awx372

**Published:** 2018-01-18

**Authors:** Ide Smets, Barnaby Fiddes, Josselyn E Garcia-Perez, Di He, Klara Mallants, Wenjia Liao, James Dooley, George Wang, Stephanie Humblet-Baron, Bénédicte Dubois, Alastair Compston, Joanne Jones, Alasdair Coles, Adrian Liston, Maria Ban, An Goris, Stephen Sawcer

**Affiliations:** 1 Laboratory for Neuroimmunology, Department of Neurosciences, KU Leuven, Belgium; 2 Department of Neurology, University Hospitals Leuven, Leuven, Belgium; 3 University of Cambridge, Department of Clinical Neurosciences, Box 165, Cambridge Biomedical Campus, Hills Road, Cambridge, CB2 0QQ, UK; 4 VIB Center for Brain and Disease Research, Leuven, Belgium; 5 Laboratory for Translational Immunology, Department of Immunology and Microbiology, KU Leuven, Belgium

**Keywords:** multiple sclerosis, susceptibility, B cells, CD40, CD86

## Abstract

The increasing evidence supporting a role for B cells in the pathogenesis of multiple sclerosis prompted us to investigate the influence of known susceptibility variants on the surface expression of co-stimulatory molecules in these cells. Using flow cytometry we measured surface expression of CD40 and CD86 in B cells from 68 patients and 162 healthy controls that were genotyped for the multiple sclerosis associated single nucleotide polymorphisms (SNPs) rs4810485, which maps within the *CD40* gene, and rs9282641, which maps within the *CD86* gene. We found that carrying the risk allele rs4810485*T lowered the cell-surface expression of CD40 in all tested B cell subtypes (in total B cells *P ≤ *5.10 × 10^−5^ in patients and ≤4.09 × 10^−6^ in controls), while carrying the risk allele rs9282641*G increased the expression of CD86, with this effect primarily seen in the naïve B cell subset (*P = *0.048 in patients and 5.38 × 10^−5^ in controls). In concordance with these results, analysis of RNA expression demonstrated that the risk allele rs4810485*T resulted in lower total CD40 expression (*P = *0.057) but with an increased proportion of alternative splice-forms leading to decoy receptors (*P = *4.00 × 10^−7^). Finally, we also observed that the risk allele rs4810485*T was associated with decreased levels of interleukin-10 (*P = *0.020), which is considered to have an immunoregulatory function downstream of CD40. Given the importance of these co-stimulatory molecules in determining the immune reaction that appears in response to antigen our data suggest that B cells might have an important antigen presentation and immunoregulatory role in the pathogenesis of multiple sclerosis.

## Teaser

Increasing evidence implicates B cells in the pathogenesis of multiple sclerosis. Smets *et al.* report that the CD40 multiple sclerosis risk allele lowers CD40 expression, whereas the CD86 risk allele increases CD86 expression. B cells may have an important antigen presentation and immunoregulatory role in multiple sclerosis.

## Introduction

Multiple sclerosis is an autoimmune disease of the central nervous system that is characterized pathologically by inflammation, demyelination and axonal loss. Genome-wide association studies have enabled the identification of 11 human leukocyte antigen (HLA) and 110 non-HLA genetic risk factors (The Australia and New Zealand Multiple Sclerosis Genetics Consortium, 2009; Patsopoulos *et al.*, 2011; The International Multiple Sclerosis Genetics Consortium and The Wellcome Trust Case Control Consortium 2, 2011; The International Multiple Sclerosis Genetics Consortium, 2013; Moutsianas *et al.*, 2015). Collectively these variants explain 28% of heritability to the disease (The International Multiple Sclerosis Genetics Consortium, 2013), but to date translating these associations into meaningful biology has proven to be difficult (Goris *et al.*, 2012; Sawcer *et al.*, 2014). The associated regions are heavily enriched for genes with known immunological function, pointing to a primary role of the adaptive immune system in the pathogenesis of the disease (The International Multiple Sclerosis Genetics Consortium and The Wellcome Trust Case Control Consortium 2, 2011; The International Multiple Sclerosis Genetics Consortium, 2013; Dendrou *et al.*, 2015). Because of their limited contribution to the induction of experimental autoimmune encephalomyelitis (EAE) in mouse models, the contribution of B cells to multiple sclerosis development has long been overshadowed by investigation into T cells. Recent data on the role of B cells in the pathogenesis of multiple sclerosis (Schubert *et al.*, 2015; Dooley *et al.*, 2016), and the highly promising results of B cell directed therapies (Hauser *et al.*, 2017; Montalban *et al.*, 2017) in the disease have challenged the earlier T cell paradigm. Several of the multiple sclerosis risk single nucleotide polymorphisms (SNPs) are located in or near genes for co-stimulatory molecules (The Australia and New Zealand Multiple Sclerosis Genetics Consortium, 2009; The International Multiple Sclerosis Genetics Consortium and The Wellcome Trust Case Control Consortium 2, 2011; The International Multiple Sclerosis Genetics Consortium, 2013), playing a role in B cells in the context of antigen presentation, such as CD40 and CD86. Current evidence suggests that most genetic risk factors for complex disease do not alter protein function, but rather exert their effects by changing the expression of genes in immune cells (Maurano *et al.*, 2012; Raj *et al.*, 2014; Sawcer *et al.*, 2014). In this study we assessed surface expression of key co-stimulatory molecules (CD40 and CD86) on B cell subtypes in patients with untreated multiple sclerosis and healthy control subjects, and correlated our findings with genotype at associated SNPs rs4810485 and rs9282641.

## Materials and methods

### Study population

All cases and controls gave written informed consent and the study was approved by the Ethics Committee of the University Hospitals Leuven (ML4733, cases) and the University of Cambridge (REC-11/33/0007, controls).

#### Cases

A study population of 68 unrelated patients of Caucasian descent fulfilling McDonald criteria for multiple sclerosis was included at the outpatient Department of Neurology of the University Hospitals Leuven. Demographic and clinical data were collected through a questionnaire and inspection of medical records (Supplementary Table 1). All patients were untreated at the time of sampling with no history of cancer, immunosuppressive treatments or antibiotics, anti-allergy or anti-inflammatory treatment in the week prior to sampling.

#### Control subjects

We studied 162 healthy volunteers recruited from the Cambridge BioResource and selected on the basis of their rs9282641 genotype. The date of attendance of the healthy controls was randomized with respect to genotype and all data collection and sample testing was undertaken fully blinded to genotype.

### Immunophenotyping

#### Leuven

Heparinized blood was collected from patients and rested at 18°C for 2–4 h prior to isolation of peripheral blood mononuclear cells (PBMCs) using lymphocyte separation medium (LSM, MP Biomedicals). PBMCs were frozen in 10% dimethyl sulfoxide (Sigma) and stored at −80°C prior to analysis. PBMCs were processed in different batches at four different time points. Cell surface expression in specific cell subtypes was examined using a B cell panel (CD19-BV510, CD27-AF-700, CD38-APC, CD24-BV421, IgM-PE, IgD-APC-Cy7, CD40-PE-Cy-7 and CD86-PE-CF594). Six B cell populations were measured using the following gating strategy: total B (CD19^+^), transitional (CD19^+^CD24^hi^CD38^hi^), naïve (CD19^+^CD27^−^), non-switched memory (CD19^+^CD27^+^IgD^+^), class-switched memory (CD19^+^CD27^+^IgD^−^) and plasmablasts (CD19^+^CD24^−^CD38^hi^). Expression of CD40 on non-B cells was performed using CD19^−^CD40^+^ cells. Based on CD40 expression patterns and forward scatter/side scatter analysis, these cells are largely myeloid in origin. Expression of CD40 and CD86 was measured as percentage of positive cells and as mean and, where appropriate mode, of fluorescence intensity (MFI) across positive cells for each cell type. Representative fluorescence-activated cell sorting (FACS) plots are shown in Supplementary Figs 1 and 2.

#### Cambridge

Heparinized venous blood was collected of healthy controls recruited from the Cambridge BioResource (http://www.cambridgebioresource.group.cam.ac.uk), all samples were collected in the morning and were fully processed within 4 h. PBMCs were isolated by density gradient centrifugation using Ficoll-Paque^™^ PLUS (GE Healthcare). Cell surface expression of CD40 and CD86 in specific cell subtypes was examined using a B cell panel (CD19-PerCP-Cy5.5, CD27-PE-Cy7, IgD-V500, CD40-FITC, and CD86-APC) defining total (CD19^+^), naïve (CD19^+^CD27^−^), non-switched memory (CD19^+^CD27^+^IgD^+^) and class-switched memory (CD19^+^CD27^+^IgD^−^) cell types. Expression of CD86 was also assessed in monocytes using a second panel (CD14-APC-H7, CD16-PE, HLA-DR-V500, CD80-FITC, and CD86-APC), defining total (CD14^+^), classical (CD14^hi^CD16^−^), intermediate (CD14^hi^CD16^+^) and non-classical (CD14^lo^CD16^+^) monocytes. Sample-specific isotype controls were determined for both CD86 and CD40. Ultimately, we were able to obtain isotype controlled data for CD86 and CD40 in B cells from 135 subjects and for CD86 in monocytes from 144 subjects. Expression of CD40 and CD86 was measured both as a percentage of cells and as MFI ratio within positive cells. The latter corresponds with the ratio between the observed geometric mean fluorescence and the geometric mean fluorescence in the corresponding sample specific isotype control. Representative FACS plots are shown in Supplementary Figs 3 and 4.

### Genotyping

SNP genotyping was undertaken using predesigned assays for the CD40 SNP rs4810485 (C_1260190_10) (cases and controls) and the CD86 SNP rs9282641 (C_30239585_20) (cases), run on a 7300 Sequence Detection Instrument or Quantstudio 7K Flex System (Thermo Fisher). For healthy controls, rs9282641 genotypes were provided by the Cambridge BioResource.

### Gene expression

#### Leuven

RNA was extracted from total PBMCs and reverse transcribed using a high-capacity cDNA reverse transcription kit (Thermo Fisher). Droplet digital PCR (Bio-Rad) was performed using 50 ng cDNA according to the manufacturer’s instructions with predesigned gene expression assays (Thermo Fisher) for total *CD40* (Hs00374176_m1, spanning exon 1-2), *CD40* without exon 6 (Hs01008251_m1, spanning exon5-7) and housekeeping gene *POLR2A* (Hs00172187_m1) or custom assays (sequence available upon request) for *CD40* without exon 5 (spanning exon 4-6) and without exon 5 and 6 (spanning exon 4-7). Relative quantity of the *CD40* splice forms versus *POLR2A* and of the alternative splice forms versus total *CD40* was measured with QuantaSoft™ v1.4 (Bio-Rad).

#### Cambridge

From each control sample, we collected CD14^+^ and CD19^+^ cells using magnetic activated cell sorting (Miltenyi Biotec); by flow cytometry we achieved purities in excess of 97% and 87%, respectively. RNA was extracted from these separated cells using TRIzol® Reagent (Life Technologies). A maximum of 1 µg of RNA was reverse transcribed using SuperScript® III First-Strand Synthesis System (Life Technologies) with a 50:50 mixture of random Oligo(dT) primers. *CD86* mRNA transcript expression was then determined using TaqMan™ on the QuantStudio™ 7K Flex System. The primers used to detect mRNA expression of transcripts (i) containing the transmembrane domain; (ii) lacking the transmembrane domain; (iii) starting with the first exon; and (iv) starting with the second exon are provided in Supplementary Fig. 5. A reference gene, TATA-box binding protein (*TBP*), was used to establish the relative expression of each transcript using the standard curve method.

### Cytokine analyses

Plasma or serum samples were collected for patients and controls and stored at −80°C for subsequent analysis. In healthy controls soluble CD86 serum levels were quantified using the sandwich enzyme linked immunosorbent assay (ELISA) with a predesigned CD86 Human ELISA Kit (Abcam) with serum diluted 1:20, and plates were read using the Bio-Rad Plate Reader model 680. In patients circulating plasma levels of B cell activation factor (BAFF) were measured using a human BAFF Quantikine® ELISA (R&D Systems), and cytokines IFN-γ, IL-10, IL-12p70, IL-2, IL-4, IL-6, IL-8 and TNF-α were quantified in plasma by electrochemiluminescence immunoassay using the V-Plex Human Proinflammatory Panel Meso Scale Discovery (MSD) plates. All MSD measurements on 68 untreated multiple sclerosis patients were done on the same 96-well plate including an eight-point standard curve (1/4 dilutions starting from 370 pg/ml, with the eighth point being blank) (Supplementary Fig. 6). Values below the detection limit (0.03 pg/ml) are set at the detection limit for analysis. For a subset of 17 untreated multiple sclerosis patients, IL-10 levels were additionally measured from a second sample obtained 3 years [standard deviation (SD) 0.5 years] previously.

### Statistical analysis

Linear regression models in R v3.3.2 and Plink v1.07 included the phenotype (immunological or gene expression variable) as function of genotype assuming an allele dosage model (number of risk alleles as 0, 1 or 2). In the Leuven study population immunophenotyping batch was taken into account as a covariate. Despite the extensive correlation between many of the assessed variables, we applied a conservative correction factor for multiple testing of 80 [based on the number of B cell and monocyte subsets considered, and the fact that both per cent positive cells and MFI (ratio) were considered for each SNP in patients and controls]. Applying this correction factor generated a corrected significance threshold *P*-value of 0.00056 for the cellular variables. In our follow-up experiments to unravel the mechanism of action (RNA expression and cytokine analysis), a nominal significance threshold (*P = *0.05) was applied.

## Results

### *CD40* and *CD86* genotype correlates with cell surface expression

In order to study the effect of multiple sclerosis risk variants mapping within the genes for *CD40* (rs4810485) and *CD86* (rs9282641) on the surface expression of these co-stimulatory molecules in B cells we collected PBMCs from 68 untreated multiple sclerosis patients and 162 healthy volunteers (Supplementary Table 1). Using flow cytometry we then defined B cell subtypes and measured the percentage of cells that were positive for each molecule together with the surface expression levels on positive cells within defined subgroups (naïve, non-switched memory, switched memory, transitional, and plasmablasts in cases, and just the first four subtypes in controls). Cases and controls were processed independently at two sites, cases in Leuven and controls in Cambridge.

We observed notable differences in the expression of these co-stimulatory receptors across the tested B cell subsets in untreated multiple sclerosis patients (Supplementary Fig. 7). Transitional B cells (CD19^+^CD24^hi^CD38^hi^) have the highest expression levels of CD40 in untreated multiple sclerosis patients, while plasmablasts (CD19^+^CD24^−^CD38^hi^) have the lowest CD40 MFI as well as the lowest percentage of CD40-positive cells. The percentage of CD86-positive cells increases with B cell activation state from transitional and naïve over memory B cells to plasmablasts. Overall, there was no net effect of the multiple sclerosis risk SNPs on the B cell subset frequencies in patients or controls (data not shown).

**Figure 1:**
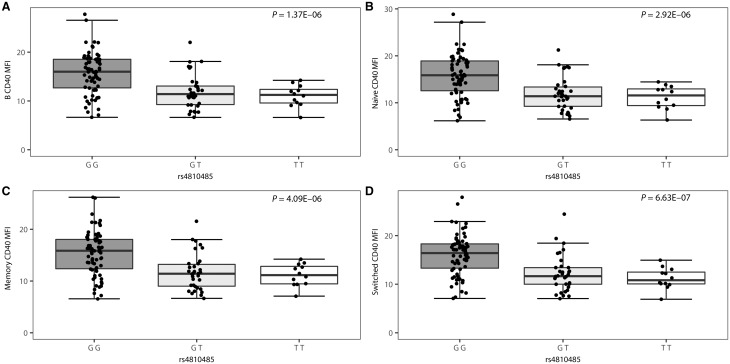
**Association of the CD40 SNP rs4810485 with B cell surface expression of CD40 in healthy controls (*n = *135).** MFI ratio of CD40 on CD40-positive cells by genotype is depicted for (**A**) B cells, (**B)** naïve B cells, (**C**) non-switched memory B cells and (**D**) class-switched memory B cells in the G/G (*n = *70), G/T (*n = *34) and T/T (*n = *12) genotype group. Box-whisker plots represent median, quartiles and 1.5× interquartile range (IQR). rs4810485*T is the multiple sclerosis risk allele. *P*-value is given for a linear regression of CD40 MFI in function of the number of T alleles.

**Figure 2:**
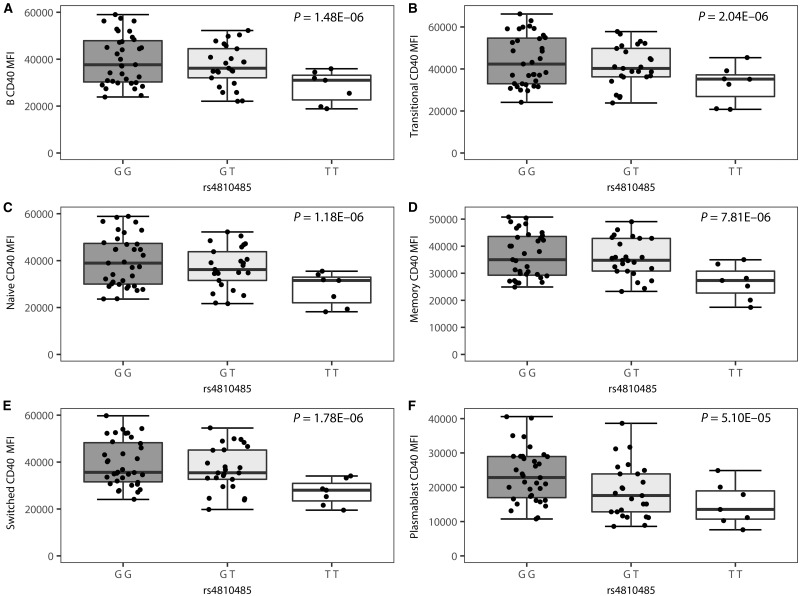
**Association of the CD40 SNP rs4810485 with B cell surface expression of CD40 in multiple sclerosis patients (*n = *66).** MFI of CD40 by genotype is depicted for (**A**) B cells, (**B**) transitional B cells, (**C**) naïve B cells, (**D**) non-switched memory B cells, (**E**) class-switched memory B cells, and (**F**) plasmablasts in the G/G (*n = *34), G/T (*n = *25) and T/T (*n = *7) genotype group. Box-whisker plots represent median, quartiles and 1.5× IQR. rs4810485*T is the multiple sclerosis risk allele. *P*-value is given for a linear regression of CD40 MFI in function of the number of T alleles including batch as covariate.

We observed highly significant associations between CD40 expression and the rs4810485 genotype in all B cell subsets (patients: *P ≤ *5.10 × 10^−5^, controls *P ≤ *4.09 × 10^−6^), with each copy of the multiple sclerosis risk allele rs4810485*T corresponding to lower CD40 cell surface expression levels (Figs 1 and 2). The percentage CD40-positive cells approximates 100% across most cell subsets with little variation, although a trend in the same direction as for MFI was observed in cases and controls (Supplementary Figs 8 and 9). In untreated multiple sclerosis patients, the myeloid cell subset showed a trend for decreased CD40 MFI (*P = *0.0024) (Supplementary Fig. 10). This association is in the same direction as for B cells, but does not survive multiple testing. Importantly, significance levels are three to four orders of magnitude smaller and the effect size is about half compared to B cells in the same study population.

**Figure 3:**
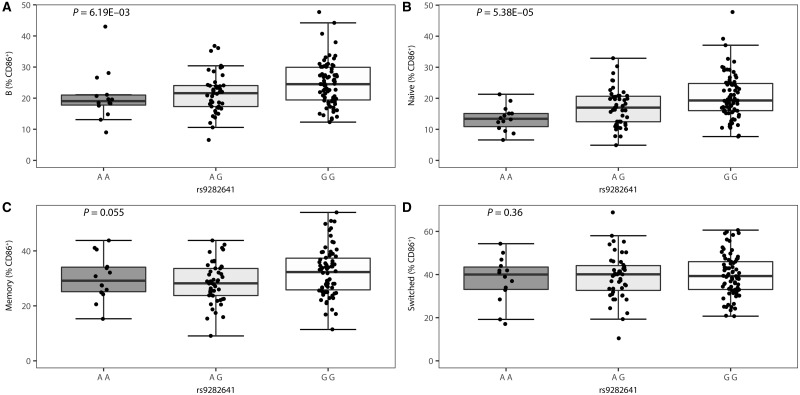
**Association of the *CD86* SNP rs9282641 with B cell surface expression of CD86 in healthy controls (*n = *135).** Percentage of CD86-positive cells by genotype is depicted for (**A**) B cells, (**B**) naïve B cells, (**C**) non-switched memory B cells and (**D**) class-switched memory B cells in the A/A (*n = *14), A/G (*n = *47) and G/G (*n = *74) genotype group. Box-whisker plots represent median, quartiles and 1.5× IQR. rs9282641*G is the multiple sclerosis risk allele. *P-*value is given for a linear regression of per cent CD86-positive in function of the number of G alleles.

**Figure 4:**
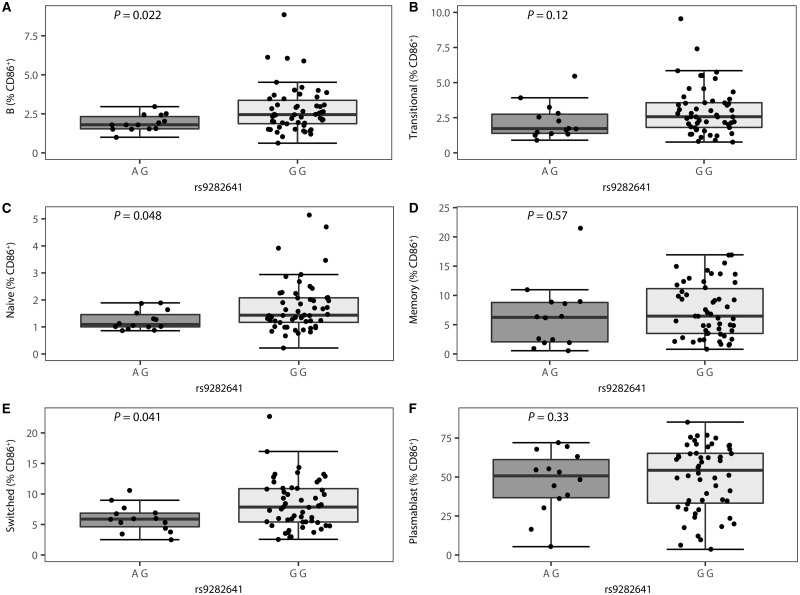
**Association of the *CD86* SNP rs9282641 with B cell surface expression of CD86 in untreated multiple sclerosis patients (*n = *67).** Percentage of CD86-positive cells by genotype is depicted for (**A**) B cells, (**B**) transitional B cells, (**C**) naïve B cells, (**D**) non-switched memory B cells, (**E**) class-switched memory B cells and (**F)** plasmablasts in the A/G (*n = *14) and G/G (*n = *53) genotype group. Box-whisker plots represent median, quartiles and 1.5× IQR. rs9282641*G is the multiple sclerosis risk allele. *P*-value is given for a linear regression of per cent CD86-positive in function of the number of G alleles with batch as covariate.

The *CD86* SNP rs9282641 has a relatively low minor allele frequency (9%), and therefore healthy controls were selected from the Cambridge BioResource on the basis of genotype in order to ensure a more uniform distribution across genotypes. In these healthy controls, we found that the multiple sclerosis risk allele rs9282641*G is associated with a higher percentage of CD86-positive B cells, primarily in naïve B cells (CD19^+^CD27^−^) (*P = *5.38 × 10^−5^) (Fig. 3). These associations are confirmed in multiple sclerosis patients, where associations were observed in three of six cell types, i.e. total B cells (CD19^+^), naïve B cells (CD19^+^CD27^−^) and class-switched memory B cells (CD19^+^CD27^+^IgD^−^) (*P ≤ *0.048) (Fig. 4). Of note, since the patients are a population-based study sample, individuals homozygous for the minor allele are few in number and the power is thereby reduced in comparison to the genotype-enriched study population of controls. Concerning CD86 MFI, nominally significant associations were observed for all B cell subsets in controls and for switched B cells in cases, but these trends did not survive correction for multiple testing (Supplementary Figs 11 and 12). As CD86 is expressed on antigen-presenting cells, including not only B cells but also monocytes, we additionally measured surface expression of CD86 in three monocyte subtypes in the healthy control individuals (classical CD14^hi^CD16^−^, non-classical CD14^lo^CD16^+^ and intermediate CD14^hi^CD16^+^) and in the myeloid subset in untreated multiple sclerosis patients. We found no evidence of association with rs9282641 genotype in any of these cell sets (Supplementary Figs 13 and 14). In the healthy control subjects we also tested for association of CD86 expression in B cells and monocytes with rs1920296 and rs2255214, two other SNPs mapping close to rs9282641 and showing independent association with multiple sclerosis (The International Multiple Sclerosis Genetics Consortium and The Wellcome Trust Case Control Consortium 2, 2011; The International Multiple Sclerosis Genetics Consortium, 2013). Again we saw no association with either SNP in any of these cell subtypes.

### *CD40* genotype correlates with total and splice-form specific RNA expression

To explore the mechanism underlying the CD40 and CD86 genotype–immunophenotype relationship further, we carried out gene expression analysis in subsets of the study population. Based on Gencode version 21 (GRCh38) the gene for *CD86* consists of eight exons, which through alternate splicing give rise to at least nine RNA transcripts (Supplementary Fig. 5), some of which lack the transmembrane domain (exon 6) and therefore give rise to soluble forms of the molecule (sCD86) (Jellis *et al.*, 1995). The known *CD86* transcripts show alternative first exon usage, and rs9282641 lies within the 5′UTR of the exon 2 starting transcripts. Hence, we measured sCD86 in serum and assessed expression at the RNA level in healthy individuals. However, we found no statistically significant association with the rs9282641*G allele for sCD86 plasma levels nor for any of the four groups of *CD86* transcripts (transmembrane exon 6-containing transcripts, soluble exon 6-skipping transcripts, exon 1-starting transcripts and exon 2-starting transcripts) in B cells or monocytes from healthy individuals (Supplementary Figs 15 and 17).

**Figure 5:**
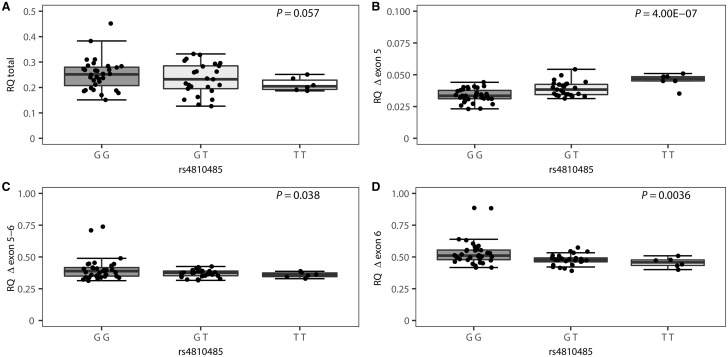
**Association of the CD40 SNP rs4810485 with total CD40 and CD40 alternative splice forms in untreated multiple sclerosis patients (*n = *65).** Genotype specific (**A**) relative quantity of total CD40 expression (spanning exons 1–2) versus housekeeping gene and relative quantity of alternative CD40 splice-forms lacking (**B**) exon 5, (**C**) exons 5–6 and (**D**) exon 6 compared to relative quantity of total CD40 expression in the G/G (*n = *34), G/T (*n = *25) and T/T (*n = *6) genotype group. Box-whisker plots represent median, quartiles and 1.5× IQR. rs4810485*T is the multiple sclerosis risk allele. *P*-value is given for a linear regression of CD40 expression levels in function of the number of T alleles.

Multiple CD40 isoforms, representing both membrane-bound and soluble proteins, have been reported (Tone *et al.*, 2001; Eshel *et al.*, 2008). Hence, we additionally measured levels of total CD40 expression as well as splice-forms lacking exon 5 and/or 6 in PBMCs from the included untreated multiple sclerosis patients. In line with flow cytometry data, the multiple sclerosis risk allele rs4810485*T shows a trend for association with lower total CD40 expression (*P = *0.057). Moreover, the risk allele differentially affects the relative quantity of each splice-form compared to total CD40 expression, with increased proportions of the splice form lacking exon 5 (*P = *4.00 × 10^−7^) and decreased proportions of the splice form lacking exon 6 (*P = *0.0036) (Fig. 5). There is a consistent association (*P* < 0.05) between higher relative expression of the CD40 isoform lacking exon 5 and lower expression of full-length CD40 protein on the cell membrane (CD40 MFI) in overall B cells and in four of five B cell subsets (Supplementary Table 2).

### *CD40* genotype correlates with downstream interleukin-10 expression

**Figure 6:**
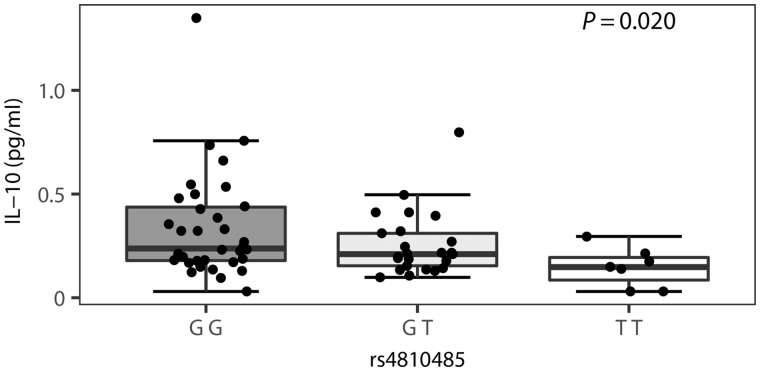
**Association of the CD40 SNP rs4810485 with serum IL-10 levels in untreated multiple sclerosis patients (*n = *66).** IL-10 levels are measured in the G/G (*n = *34), G/T (*n = *25) and T/T (*n = *7) genotype group. Box-whisker plots represent median, quartiles and 1.5× IQR. rs4810485*T is the multiple sclerosis risk allele. *P*-value is given for a linear regression of IL-10 levels in function of the number of T alleles.

Finally, to investigate the downstream consequences of the observed genotype–phenotype correlations, we assessed a panel of cytokines in plasma samples from multiple sclerosis patients. No significant association was observed between the CD86 rs9282641 genotype and cytokine plasma levels (*P* ≥ 0.25). CD40 genotype on the other hand was associated with interleukin-10 (IL-10) plasma levels. The IL-10 assay has a dynamic range of 0.03–233 pg/ml, a sensitivity of 0.03 pg/ml, and an r^2^ > 0.99 across technical replicates. For a subset of *n* = 17 untreated multiple sclerosis patients, IL-10 levels were measured from samples at two different time points, with an average interval of 3 years. Correlation amongst these different time-point measures in the same individuals resulted in an r^2^ = 0.67, indicating relative intra-individual stability of IL-10 plasma levels over long periods of time (Supplementary Fig. 18). This is in line with the high level of interindividual variation, with low longitudinal variation, which we previously demonstrated for cellular immune phenotypes (Carr *et al.*, 2016). The multiple sclerosis risk allele rs4810485*T, associated with lower CD40 surface expression levels in the flow cytometry data, corresponded to lower IL-10 levels (Fig. 6).

## Discussion

By applying detailed immunophenotyping in untreated multiple sclerosis patients and healthy controls, we have demonstrated that cell surface expression of the co-stimulatory molecules CD40 and CD86 on B cells is influenced by local genetic variants associated with disease susceptibility, suggesting that B cells contribute to the pathogenesis of the disease. The fact that the effect of rs4810485 on the expression of CD40 is seen in all B cell subsets, while the effect of rs9282641 on CD86 expression is essentially confined to naïve cells, might reflect involvement of a ubiquitous enhancer for CD40 expression and an enhancer with more restricted cell type specificity for CD86.

Our data in untreated patients and controls indicate a strong genotype–phenotype correlation for CD40 across B cell differentiation stages (transitional, naïve, non-switched memory, switched memory and plasmablasts, with *P*-values ranging from 10^−5^ to 10^−7^) and hence extend a previously reported association reaching nominal significance in overall B cells from 86 healthy controls (Field *et al.*, 2015). In both datasets, the multiple sclerosis risk allele rs4810485*T is associated with decreased cell surface expression. Previous analysis of *CD40* expression at the RNA level has generated contradictory results (Jacobson *et al.*, 2005; Onouchi *et al.*, 2012; Field *et al.*, 2015); however, by using sensitive droplet digital PCR to measure the levels of alternative splice forms, we here demonstrate that transcripts are differentially affected by genotype. In PBMCs from untreated multiple sclerosis patients, rs4810485*T is associated with a lower proportion of the splice form lacking exon 6, in line with findings in B cell lines derived from healthy controls (Onouchi *et al.*, 2012), and a higher proportion of the splice form lacking exon 5. The alternative splice-forms lacking exon 6 and exon 5 encode soluble decoy receptors: the first one has antagonistic properties whereas the second isoform has been reported not to bind CD40 or act downstream of CD40-CD40L stimulation, but may bind other CD40 ligands (Eshel *et al.*, 2008). During B cell activation and *CD40* upregulation, pre-*CD40* mRNA splicing is shifted from predominantly generating the full-length isoform to reaching higher proportions of the antagonistic soluble form and this process is thought to provide a negative feedback-loop (Tone *et al.*, 2001). Hence, *CD40* genetic variation affecting a ubiquitous CD40 enhancer may at the same time induce this post-transcriptional regulation process affecting splicing.

Notably, the *CD40* SNP is shared across autoimmune diseases but with opposite effects: the rs4810485*T allele increases the risk of multiple sclerosis (The Australia and New Zealand Multiple Sclerosis Genetics Consortium, 2009; Patsopoulos *et al.*, 2011; The International Multiple Sclerosis Genetics Consortium and The Wellcome Trust Case Control Consortium 2, 2011; The International Multiple Sclerosis Genetics Consortium, 2013) and inflammatory bowel disease (Jostins *et al.*, 2012) but is protective against rheumatoid arthritis (Raychaudhuri *et al.*, 2008), systemic lupus erythematosus (Vazgiourakis *et al.*, 2011), autoimmune thyroid disease (Tomer *et al.*, 2002) and Kawasaki disease (Onouchi *et al.*, 2012). CD40 is important for T cell dependent immunoglobulin class-switching and germinal centre formation (Kawabe *et al.*, 1994) and plasmablast growth and differentiation (Garcia de Vinuesa *et al.*, 1999). Hence, increased CD40 expression levels on B cell subsets involved in antibody production, and in particular the effect on both expression levels and the percentage of CD40-positive plasmablasts that we report for the first time, are consistent with increased susceptibility to autoimmune diseases involving a strong humoral response (rheumatoid arthritis; systemic lupus erythematosus; autoimmune thyroid disease and Kawasaki disease). On the other hand, CD40 ligation has beneficial effects including the induction of regulatory T cells (Guiducci *et al.*, 2005) and the formation and function of IL-10 producing B cells with immunomodulatory properties (Yanaba *et al.*, 2009). In the animal model for multiple sclerosis, EAE, the transfer of regulatory IL-10 producing B cells (B10) into *Cd19*^−/−^ B cell deficient mice reduces EAE severity and reduces autoantigen-specific CD4^+^ T cell proliferation and pro-inflammatory cytokine production (Yoshizaki *et al.*, 2012). However, B10 cells that are CD40 deficient (*Cd40*^−/−^) do not reduce severity, proliferation or cytokine production. On the other hand, CD40 stimulation increases the number of B10 cells (Yoshizaki *et al.*, 2012). A beneficial role for regulatory B cells has also been described in humans for the two autoimmune diseases where reduced CD40 increases susceptibility, namely multiple sclerosis and inflammatory bowel disease (Li *et al.*, 2015, Dooley *et al.*, 2016). Our current work indeed pinpoints to the regulatory function of CD40, through downstream IL-10 production, as mechanism of action underlying this association, with the risk allele leading to lower CD40 cell surface expression and lower IL-10 levels.

Our data on expression levels of the CD86 co-stimulatory molecule across B cell subsets, from low percentages of positive cells amongst the naïve B cell subset increasing over memory B cells to highest levels in plasmablast reflect B cell activation (Liu *et al.*, 1995; Tangye *et al.*, 1998; Ellyard *et al.*, 2004; Good *et al.*, 2009). CD86 expression levels on B cells are increased in the CSF compared to the peripheral blood of multiple sclerosis patients (Fraussen *et al.*, 2016), possibly reflecting the high expression levels on plasmablasts. We demonstrate that the local multiple sclerosis risk allele rs9282641*G is associated with a higher percentage of CD86-positive B cells in untreated multiple sclerosis patients and controls. The effect appears strongest in a specific B cell subset, naïve B cells, again for both patients and controls. Higher CD86 expression in this B cell subset has previously been associated with multiple sclerosis versus controls (Niino *et al.*, 2009; Fraussen *et al.*, 2016) with relapse versus remission in multiple sclerosis (Niino *et al.*, 2009), and with a phenotype of high neurodegeneration in multiple sclerosis (Comabella *et al.*, 2016). Quite how and why changes in the proportion of naïve B cells expressing CD86 influence the development of the disease is not clear. Such a change might simply reflect an enhanced tendency towards activation or perhaps influence an individual’s ability to generate regulatory T cells (Reichardt *et al.*, 2007). Given that the expression of CD86 is likely to be essential for B cells undertaking an antigen presentation role, another possible interpretation of these findings is that the multiple sclerosis associated genotype increases the proportion of B cells undertaking this function, and that perhaps antigen presentation contributes to pathogenesis. It is well established that many of the genes implicated through genetic analysis of multiple sclerosis are involved in the signalling between antigen presenting cells and lymphocytes, and it is notable that these associations are generally stronger for the receptor/ligand from the antigen presenting cell side of each of such interaction (Sawcer *et al.*, 2014). This is most evident in the comparison between the very profound class II MHC associations in multiple sclerosis (Moutsianas *et al.*, 2015) and the near absence of any notable association with variation in the regions of the T cell receptor genes. Within a single genome-wide association study such differences might simply be assumed to reflect the inevitable random variation in the effect sizes measured for relevant SNPs. However, the fact that these differences have been reproduced in large follow-up efforts, such as the immunochip study (The International Multiple Sclerosis Genetics Consortium, 2013) raises the possibility that the differences genuinely reflect the relative importance of antigen presenting cells in the aetiology of multiple sclerosis.

Our study design combined two independent approaches with different workflows and methodologies each with their own strengths and limitations. One approach focused on the extensive investigation of all B cell subsets whereas the other invested heavily in methodological control of the flow cytometry and considered a wider range of potentially relevant genetic variation. Importantly, concordant results and conclusions were reached in both study populations, independent of the experimental design, providing mutual replication for these results.

Limitations of our *ex vivo* study include the inability to directly correlate the genotype-dependent *CD40* RNA and protein expression to the increased IL-10 production. Studies in the animal model of multiple sclerosis, EAE, have shown that CD40 ablation or stimulation regulates IL-10-producing B cell number and function, as described above. Demonstrating the same effects for the more subtle genotype-dependent expression differences in humans would require a follow-up study with the development of an *in vitro* model that has the capacity to detect B cell stimulation effects in the physiological range of SNPs, possibly through an agonistic CD40 antibody or co-culture with a T cell line expressing CD40 ligand (CD154).

Our data indicating that the studied multiple sclerosis risk SNPs affect the expression of co-stimulatory molecules on B cells have implications for treatment strategies in multiple sclerosis. Existing treatments such as interferon-beta and fingolimod affect CD86 expression in different ways (Niino *et al.*, 2009; Fraussen *et al.*, 2016)—in line with the general opposing effects of these treatments on the peripheral immune system (Dooley *et al.*, 2016). However, the recently completed ACCLAIM clinical trial with abatacept, blocking CD80-CD86 co-stimulation and approved for rheumatoid arthritis and juvenile idiopathic arthritis, failed in multiple sclerosis (Khoury *et al.*, 2017). As both our genotype data and data on existing treatments (Niino *et al.*, 2009) indicate cell-subset-specific effects on CD86 expression in multiple sclerosis, in particular related to naïve B cells, such subset-specific effects may underlie this unexpected outcome. Phase II trials blocking the CD40-CD40L pathway—currently halted due to thromboembolic events (Summers deLuca and Gommerman, 2012)—were initiated on the basis of a beneficial role of CD40 blockade in EAE (Gerritse *et al.*, 1996; Howard *et al.*, 1999), accompanied in particular by a reduction in anti-MOG antibodies (Boon *et al.*, 2001). These animal model data are in line with the higher-expression allele increasing risk of autoimmune diseases with a strong humoral response. Our data in humans, however, suggest that CD40 blockade may be counter-productive in multiple sclerosis as it may inhibit beneficial mechanisms such as IL-10 producing immunomodulatory B cells. We have previously demonstrated the upregulation of BAFF and transitional B cells as a unique common pathway of current efficacious multiple sclerosis treatments, shedding new light on the failure of anti-BAFF trials, which also appeared promising in EAE (Dooley *et al.*, 2016). Together, these data highlight the failure of the EAE model to mimic the B cell component of multiple sclerosis and indicate how human genotype–immunophenotype data may direct the development of therapeutic strategies.

## Supplementary Material

Supplementary Data

## Abbreviations

EAE: experimental autoimmune encephalomyelitis
MFI: mean fluorescence intensity
PBMC: peripheral blood mononuclear cells
SNP: single nucleotide polymorphism
