# Supplementary material for: Multiple sclerosis risk variants alter expression of co-stimulatory genes in B cells
Source: Brain. 2018 Jan 18;141(3):786–96. doi: 10.1093/brain/awx372 (PMC5837558; doi:10.1093/brain/awx372)

Supplementary Table 1. Study population

Demographical/clinical characteristics	Multiple sclerosis patients
Number of patients included	68
Gender: N female/male (% female)	50/18 (74%)
Disease course: Bout onset/Progressive (% bout onset)	54/12 (79%)
Age at onset (years): median (range)	34 (17-68)
Age at sampling (years): median (range)	52 (23-78)
Disease duration (years): median (range)	13 (0-44)
MSSS at sampling: median (range)	1.76 (0.11-9.13)

Supplementary Table 2. Association results between CD40 isoforms and CD40 cell surface expression in B cell subsets of untreated multiple sclerosis patients. Linear regression was performed with the different CD40 spliceforms as function of CD40 mean fluorescence intensity (MFI) taking immunophenotyping batch into account as a covariate. Significant P values are underlined. (RQ = relative quantity, ▲ = CD40 without)

CD40 MFI on the following celtypes:		RQ CD40 Total	RQ ▲ exon5	RQ ▲ exon56	RQ▲ exon6
B	Bèta	2.44e-06	-2.40e-07	1.20e-06	2.41e-06
	P	0.22	0.035	0.34	0.12
Transitional	Bèta	1.77e-06	-2.20e-07	8.87e-07	2.07e-06
	P	0.34	0.041	0.46	0.15
Naive	Bèta	2.35e-06	-2.42e-07	1.27e-06	2.42e-06
	P	0.22	0.030	0.30	0.11
Memory	Bèta	2.62e-06	-2.68e-07	8.39e-07	2.04e-06
	P	0.26	0.050	0.58	0.27
Switched	Bèta	2.10e-06	-2.17e-07	7.97e-07	2.33e-06
	P	0.27	0.053	0.52	0.12
Plasmablast	Bèta	2.13e-06	-2.02e-07	8.0e-07	2.35e-06
	P	0.30	0.10	0.56	0.16

Supplementary Figure 1. B cell flow cytometry gating strategy in untreated MS patients (Leuven). A. Lymphocyte gate selected on side scatter (SS-A) vs. forward scatter (FS-A). **B.** Single cell gated on forward scatter width vs. area. **C.** $CD19^+$ gate selected on single cells. **D.** $CD40^+$ and **E.** $CD86^+$ gate selected on B cells. **F.** Transitional B cell and plasmablast gate selected on CD38 vs. CD24 of B cells. **G. I.** $CD40^+$ and **H. J.** $CD86^+$ gate selected on respectively transitional B cells and plasmablasts. **K.** Naïve B cell gate selected on CD27 of B cells. **L.** $CD40^+$ and **M.** $CD86^+$ gate selected on naïve B cells. **N.** Memory and switched B cell gate selected on IgD and CD27. **O. Q.** $CD40^+$ and **P. R.** $CD86^+$ gate selected on respectively memory and switched B cells.

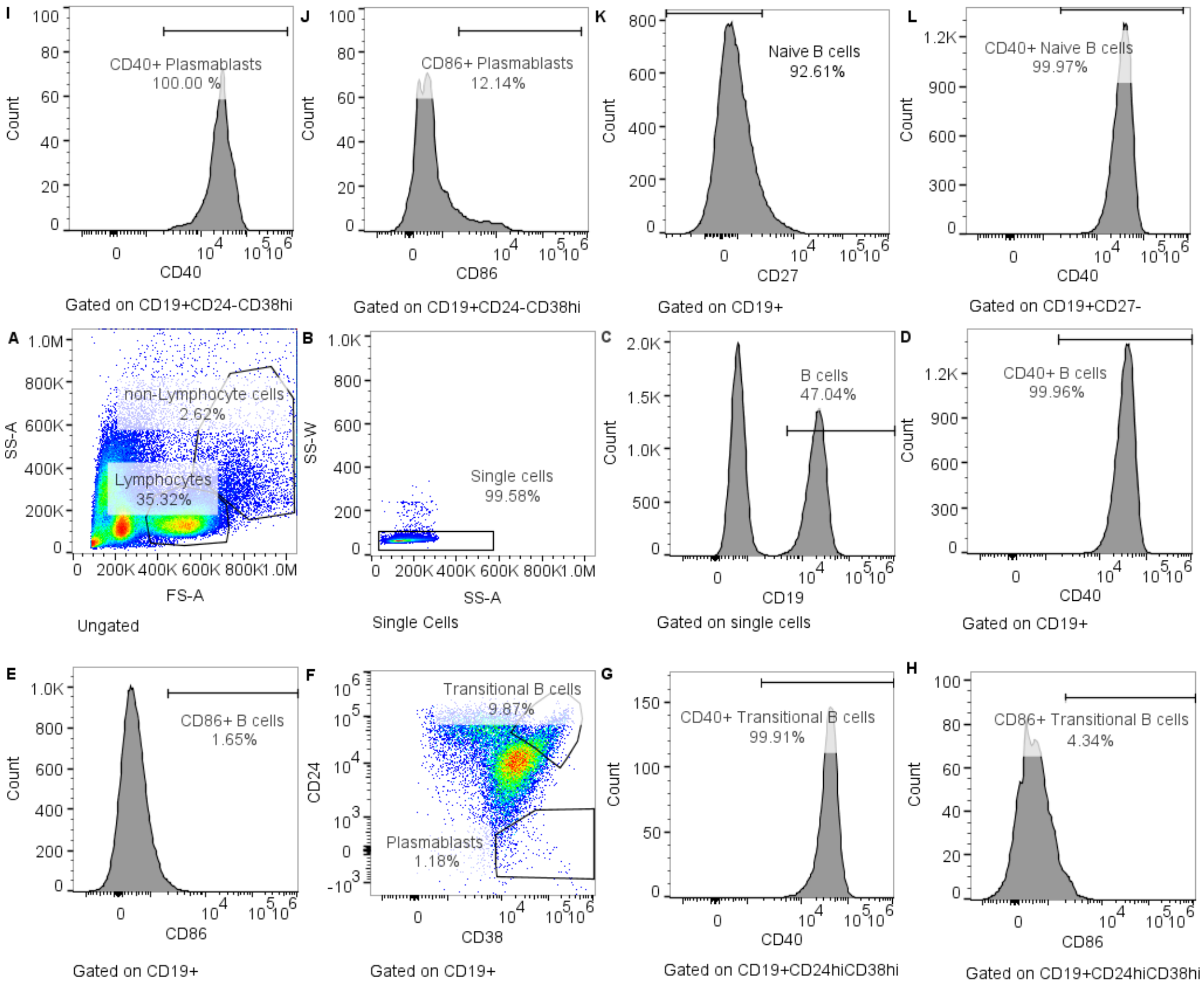
I
CD40+ Plasmablasts
100.00 %
Count
CD40
Gated on CD19+CD24-CD38hi
J
CD86+ Plasmablasts
12.14%
Count
CD86
Gated on CD19+CD24-CD38hi
K
Naive B cells
92.61%
Count
CD27
Gated on CD19+
L
CD40+ Naive B cells
99.97%
Count
CD40
Gated on CD19+CD27-
A
non-Lymphocyte cells
2.62%
Lymphocytes
35.32%
SS-A
FS-A
Ungated
B
Single cells
99.58%
SS-W
SS-A
Single Cells
C
B cells
47.04%
Count
CD19
Gated on single cells
D
CD40+ B cells
99.96%
Count
CD40
Gated on CD19+
E
CD86+ B cells
1.65%
Count
CD86
Gated on CD19+
F
Transitional B cells
9.87%
Plasmablasts
1.18%
CD24
CD38
Gated on CD19+
G
CD40+ Transitional B cells
99.91%
Count
CD40
Gated on CD19+CD24hiCD38hi
H
CD86+ Transitional B cells
4.34%
Count
CD86
Gated on CD19+CD24hiCD38hi

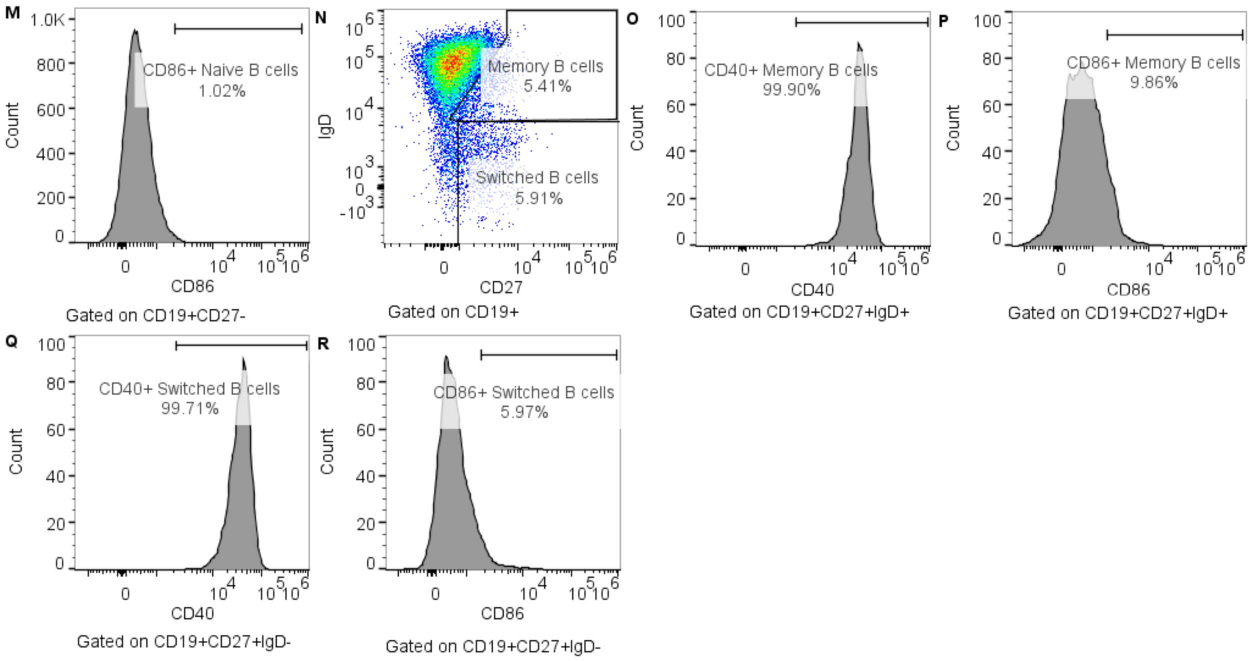
M
CD86+ Naive B cells
1.02%
Count
CD86
Gated on CD19+CD27-
N
Memory B cells
5.41%
Switched B cells
5.91%
IgD
CD27
Gated on CD19+
O
CD40+ Memory B cells
99.90%
Count
CD40
Gated on CD19+CD27+IgD+
P
CD86+ Memory B cells
9.86%
Count
CD86
Gated on CD19+CD27+IgD+
Q
CD40+ Switched B cells
99.71%
Count
CD40
Gated on CD19+CD27+IgD-
R
CD86+ Switched B cells
5.97%
Count
CD86
Gated on CD19+CD27+IgD-

Supplementary Figure 2. **Myeloid flow cytometry gating strategy in untreated MS patients (Leuven).** Expression of CD40 and CD86 on non-B cells was performed based on **A.** side scatter (SS-A) vs. forward scatter (FS-A). **B.** CD19$^-$ gate, **C.** CD40$^+$ and **D.** CD86$^+$ gate. Based on these expression patterns, these cells are largely myeloid in origin.

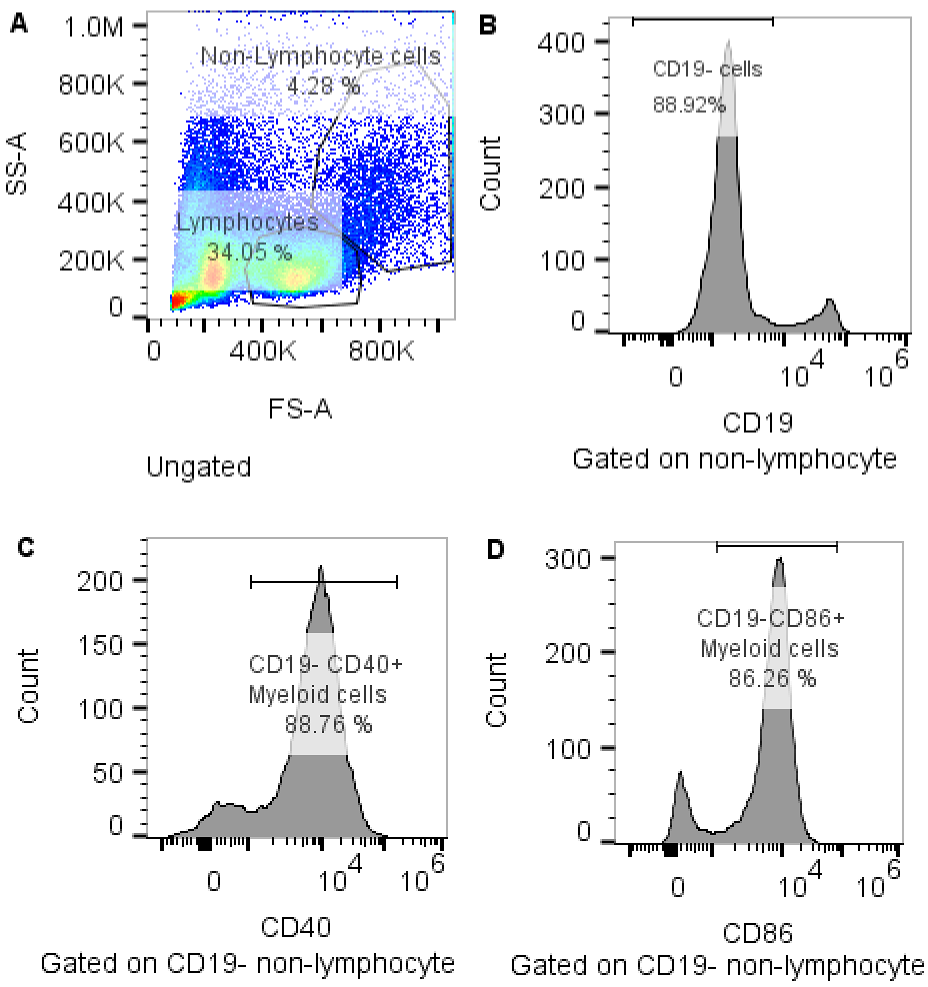

Supplementary Figure 3. B cell flow cytometry gating strategy in healthy controls (Cambridge). A. Lymphocyte gate selected on side scatter (SSC-A) vs. forward scatter (FSC-A). **B.** Single cell gated on forward scatter width vs. area. **C.** CD19$^+$, CD86$^-$, and CD86$^+$ gates selected on CD19 vs CD86, confirmed by isotype and fluorescence minus one (FMO) control samples (not shown). **D.** B cell subtypes selected using CD27 vs IgD plot of CD19$^+$ cells, confirmed by FMO controls for IgD and CD27 (not shown).

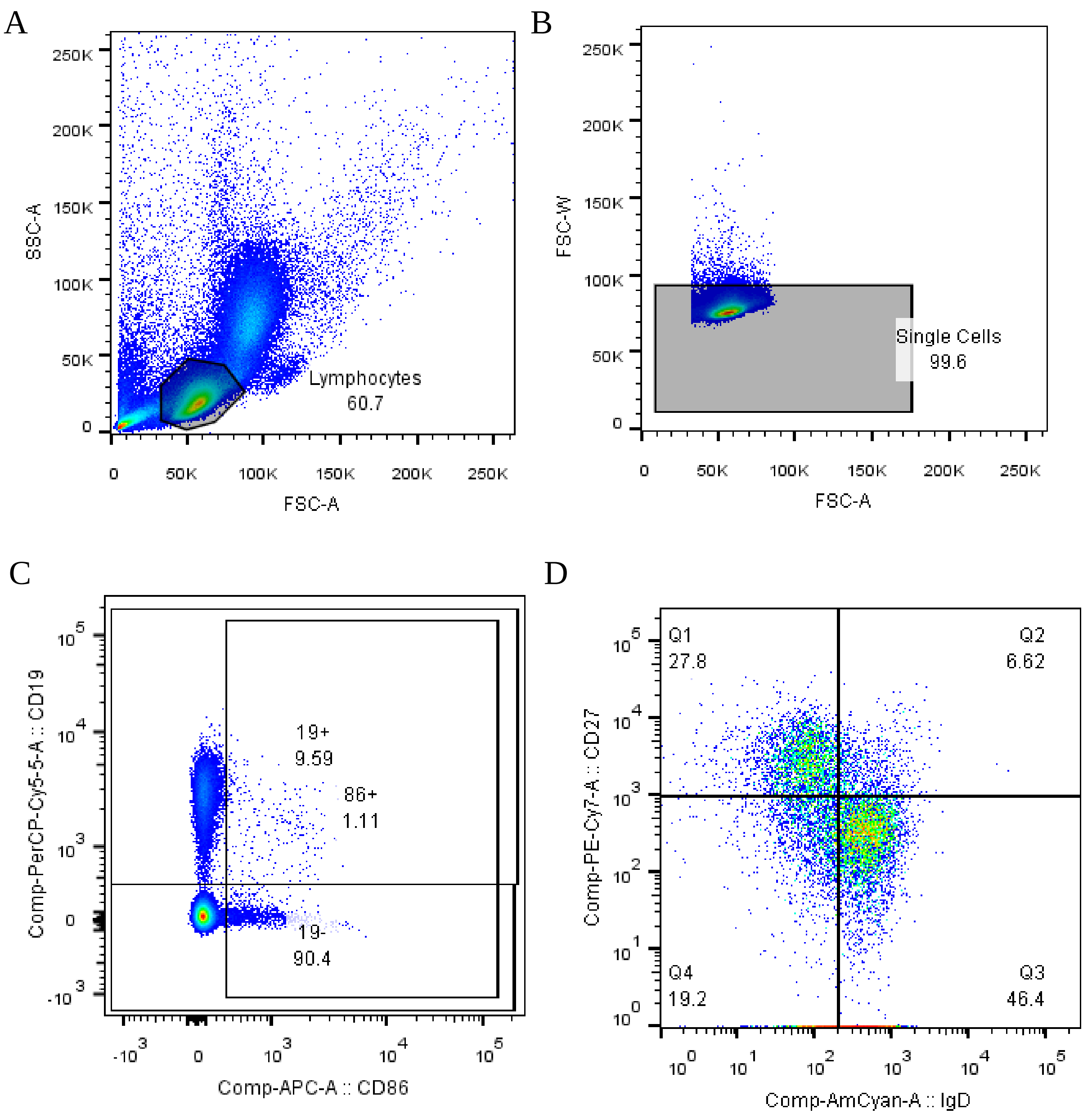

Supplementary Figure 4. **Monocyte flow cytometry gating strategy in healthy controls (Cambridge). A.** Monocyte gate selected on side scatter (SSC-A) vs. forward scatter (FSC-A). **B.** Single cell gated on forward scatter width vs. area. **C.** CD14^{+} gate selected on CD14 vs. CD16, confirmed by isotype and fluorescence minus one (FMO) control samples (not shown). **D.** Monocyte subtypes gated as shown on CD14 vs. CD16, confirmed by isotype and FMO controls (not shown).

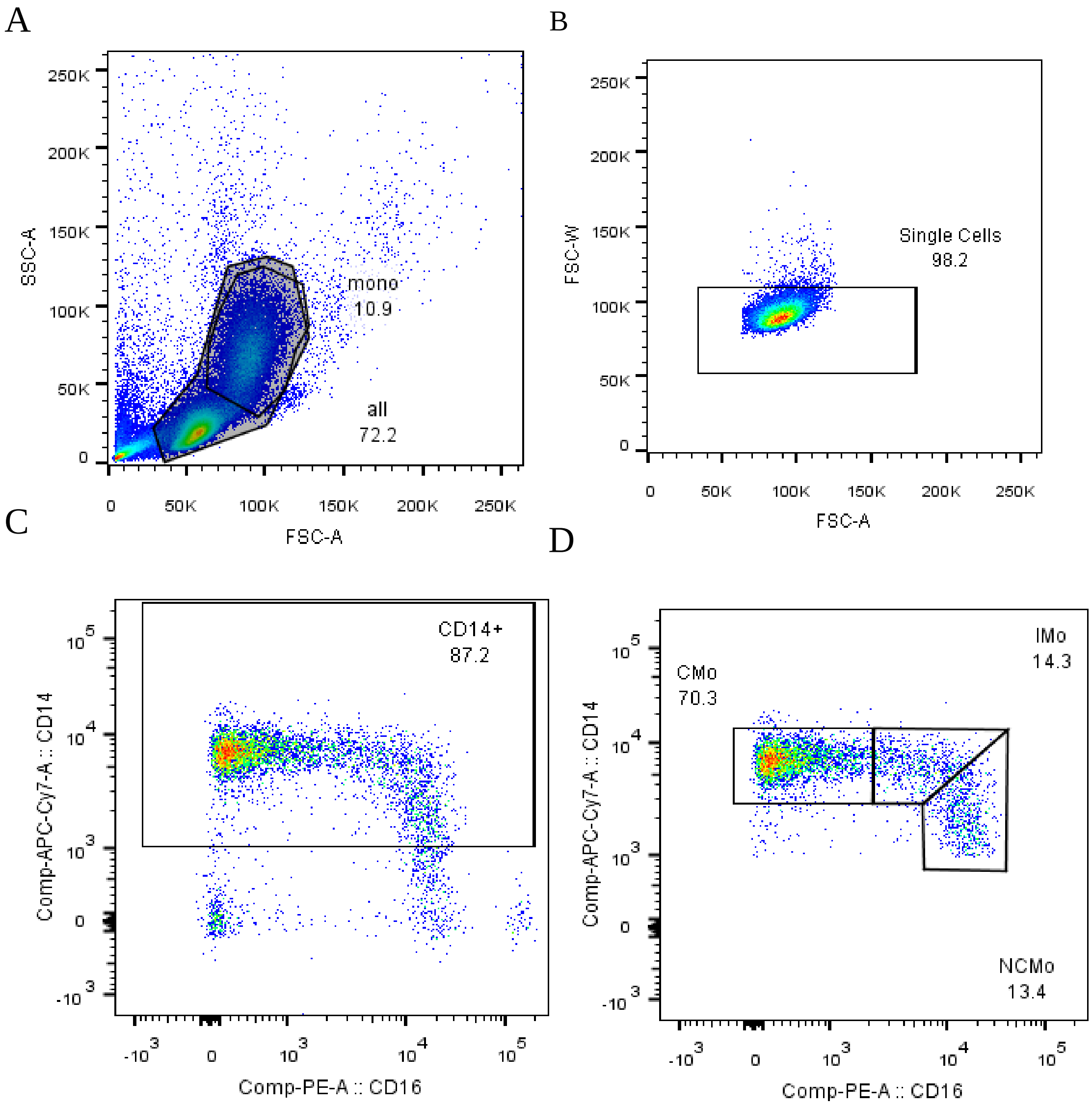

Supplementary Figure 5. Schematic of known CD86 transcripts and corresponding protein isoforms. CD86 alternative spliceforms starting with an exon 1 and some with an exon 2, some with a transmembrane (TM) domain and some without. The position of CD86 rs9282641 is shown by the vertical dashed line. Transcripts are based on Gencode version 21 (GRCh38). Primers used for particular sets of isoforms are shown below.

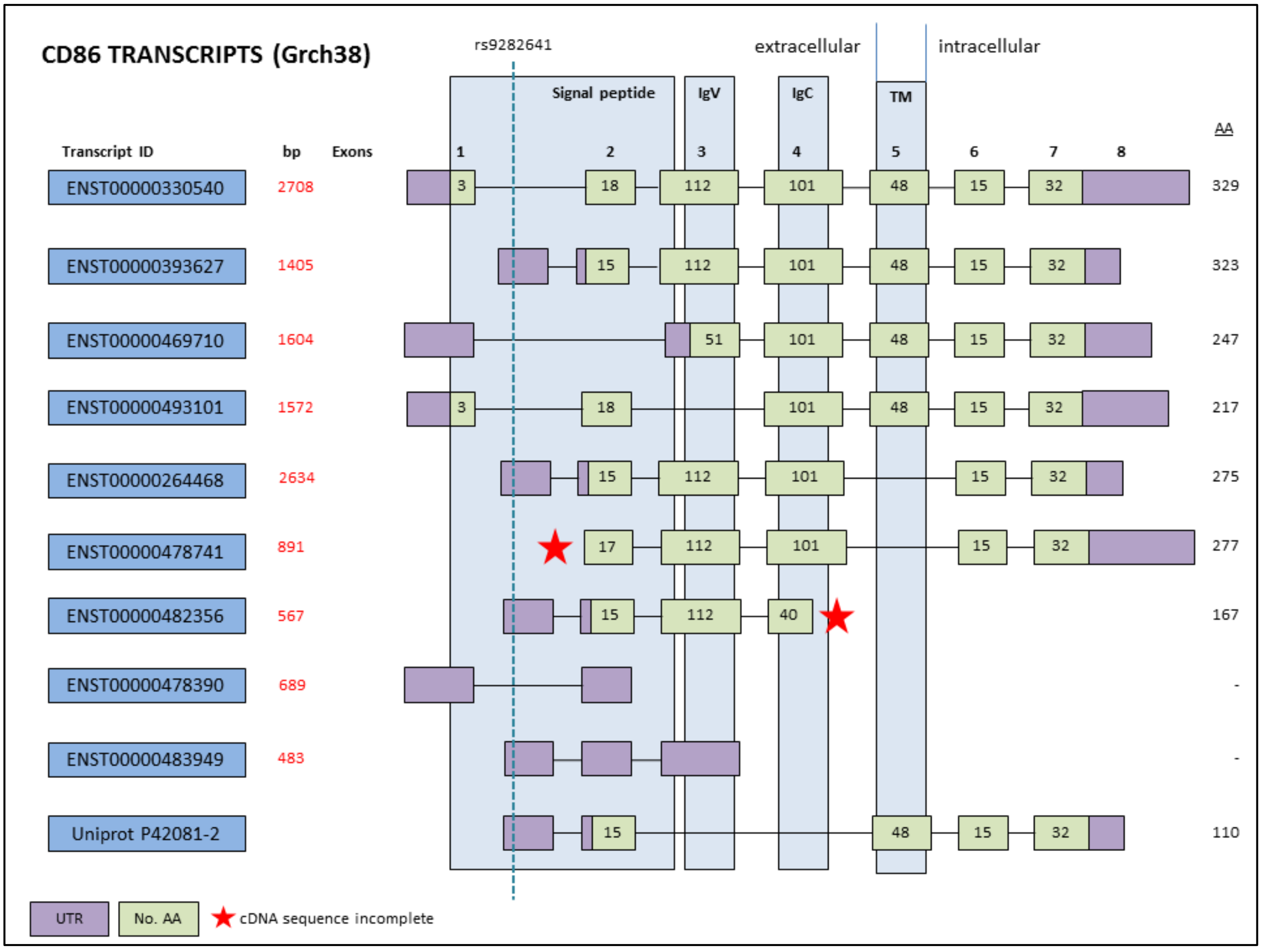

We used the following primers to quantify these 4 groups of transcripts:

<u>Applied Biosystems Assays-by-Design</u>

**** Exon 1 starting mRNA transcripts***

Forward primer	CACAGGGTGAAAGCTTTGCTT
Reverse primer	TCTGGAAATGACCCCACTCATC
Probe (FAM)	TCTGCTGCTGTAACAGG

Exon 2 starting mRNA transcripts

Forward primer	CCACCTTGCTTCTGTGTTCCTT
Reverse primer	TGTAGCTCCAAAAAGAGACCAGATG
Probe (FAM)	ATGCTGCTGTGCTTAT

Soluble mRNA transcripts (lacking transmembrane domain)

Forward primer	ACGAGCAATATGACCATCTTCTGTAT
Reverse primer	TGGTTCCTATAGAGAAAGGTGAAGATAA
Probe (FAM)	AACTGACAAGACGCGGC

<u>Applied Biosystems Assays-on-Demand</u>

Transmembrane CD86 mRNA transcripts

Hs01567025_m1

TBP (reference gene)

Hs00427620_m1

Supplementary Figure 6. Standard curve IL-10 measurement. For each plate eight standard points were included in duplicate to generate a curve by which the concentration of the unknown samples is calculated. For IL-10 the highest concentration is 370 pg/mL and 1:4 dilutions are made with the eighth point being blank. R square between duplicate measurements of the standard curve was 0.99.

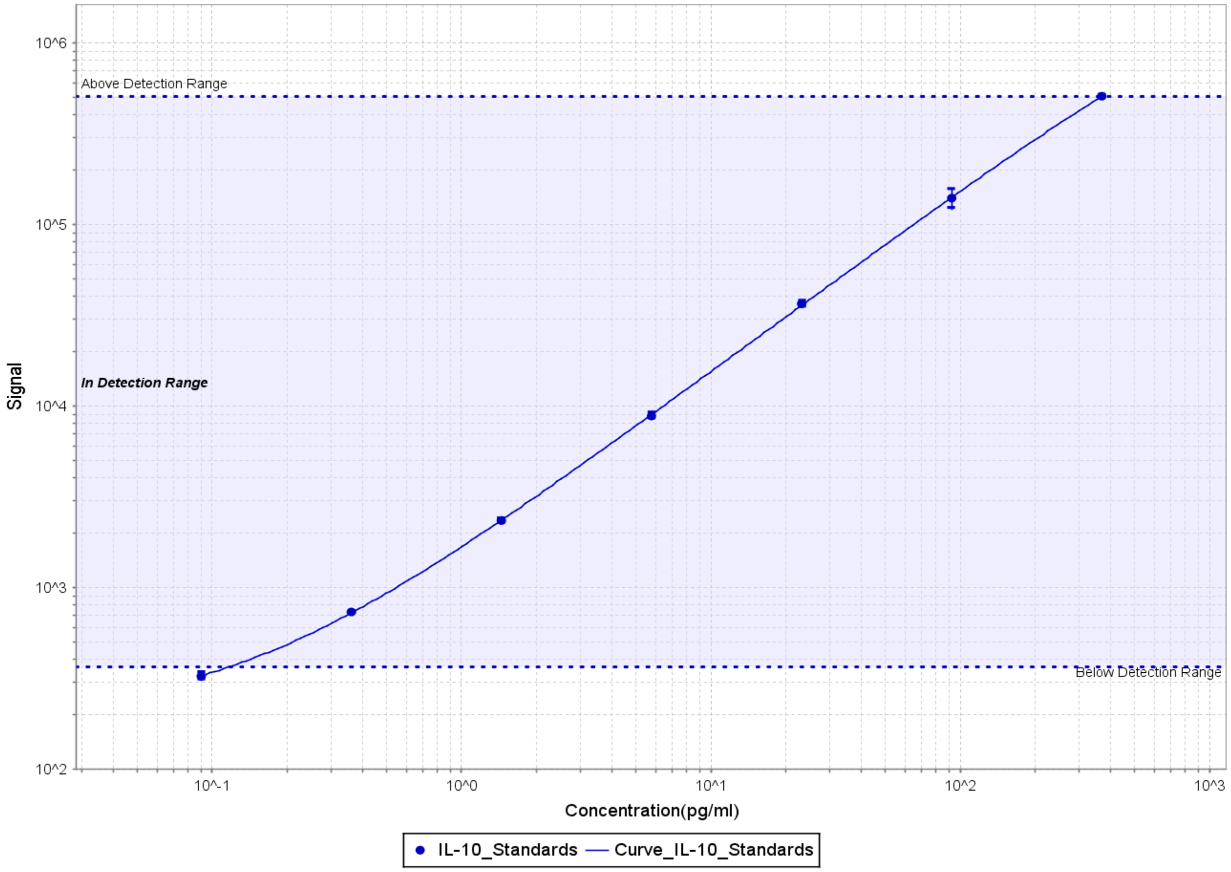

Supplementary Figure 7. Expression of co-stimulatory molecules on B cell subsets in untreated multiple sclerosis patients. A. Percentage positive cells and **B.** mean fluorescent intensity. Mean and standard deviation are given.

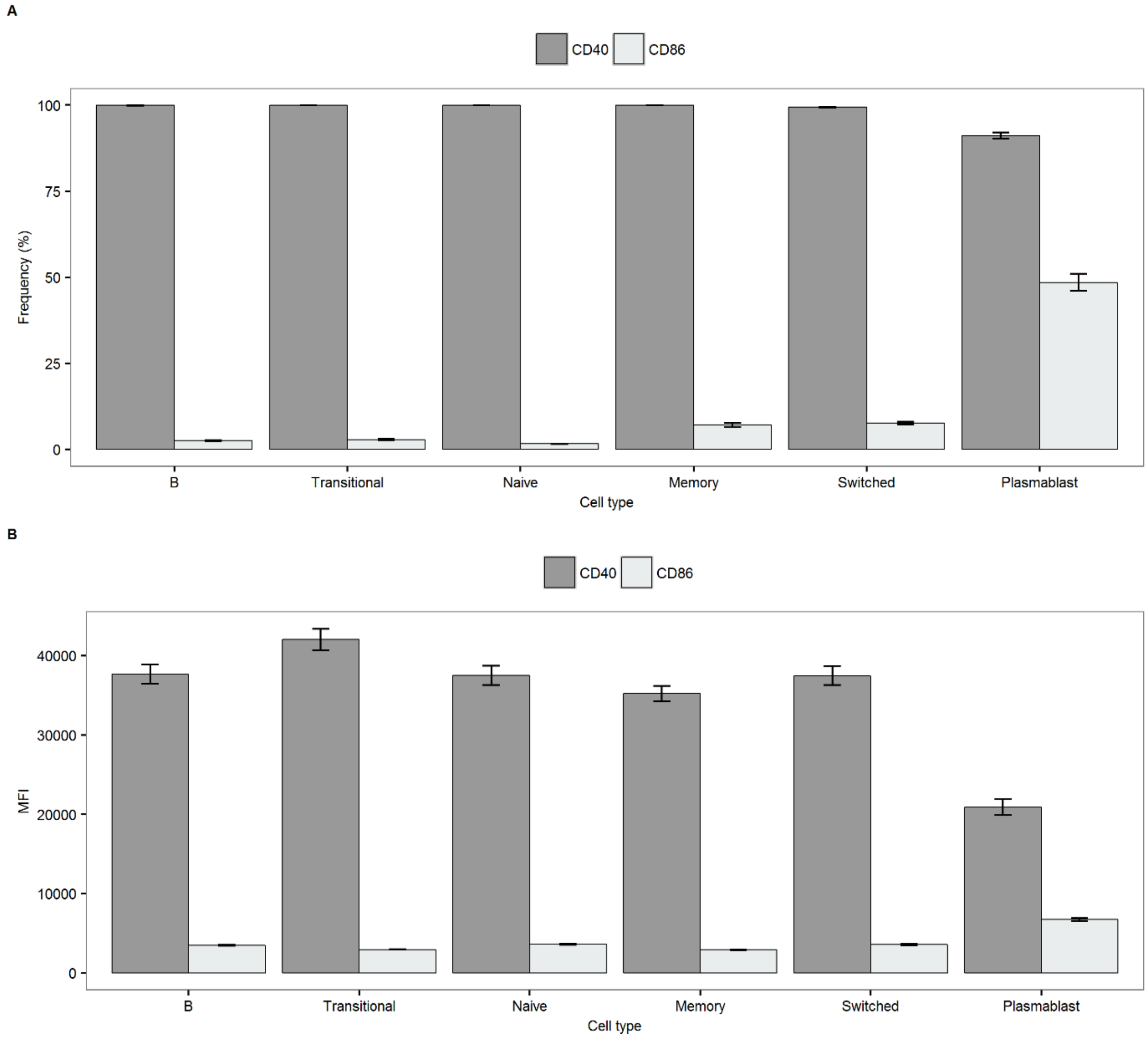

Supplementary Figure 8. Association of the CD40 SNP rs4810485 with B cell surface expression of CD40 in healthy controls. Percentage of CD40 positive cells by genotype is depicted for **A.** B cells, **B.** naïve B cells, **C.** non-switched memory B cells and **D.** class-switched memory B cells. Box-whisker plots represent median, quartiles and 1.5x interquartile range. rs4810485*T is the multiple sclerosis risk allele.

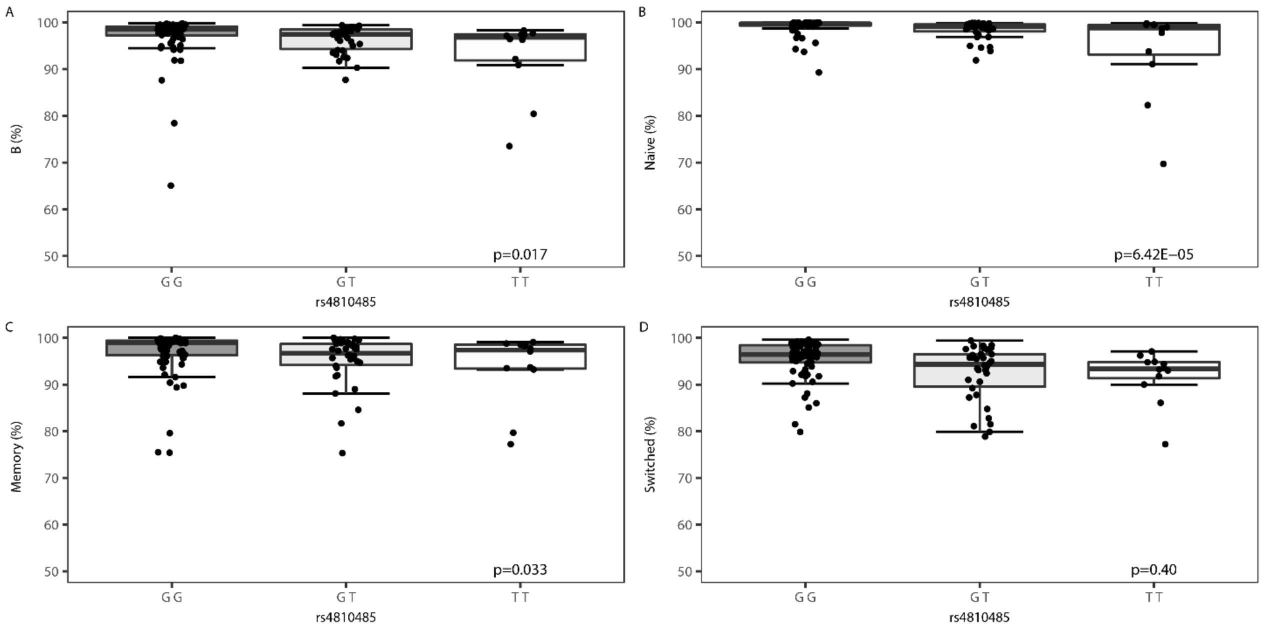

Supplementary Figure 9. Association of the CD40 SNP rs4810485 with B cell surface expression of CD40 in untreated multiple sclerosis patients. Percentage of CD40 positive cells by genotype is depicted for **A.** B cells, **B.** transitional B cells, **C.** naïve B cells, **D.** non-switched memory B cells, **E.** class-switched memory B cells and **F.** plasmablasts. Box-whisker plots represent median, quartiles and 1.5x interquartile range. rs4810485*T is the multiple sclerosis risk allele.

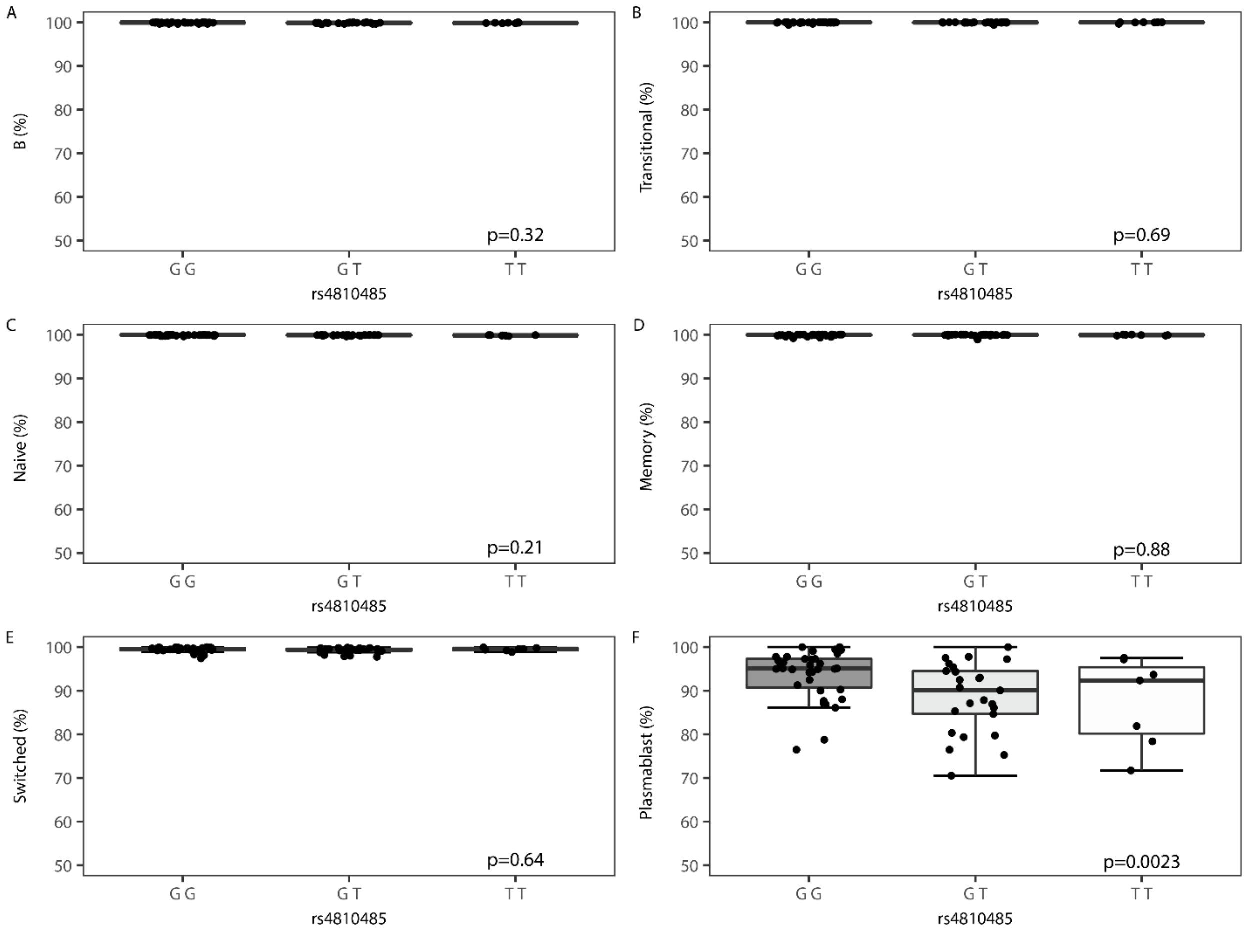

Supplementary Figure 10. Association of the CD40 SNP rs4810485 with myeloid subset surface expression of CD40 in untreated multiple sclerosis patients. A. Mean and **B.** mode of fluorescent intensity of CD40 by genotype is depicted in $CD40^{+}$ $CD19^{-}$ myeloid cells. Box-whisker plots represent median, quartiles and 1.5x interquartile range. rs4810485*T is the multiple sclerosis risk allele.

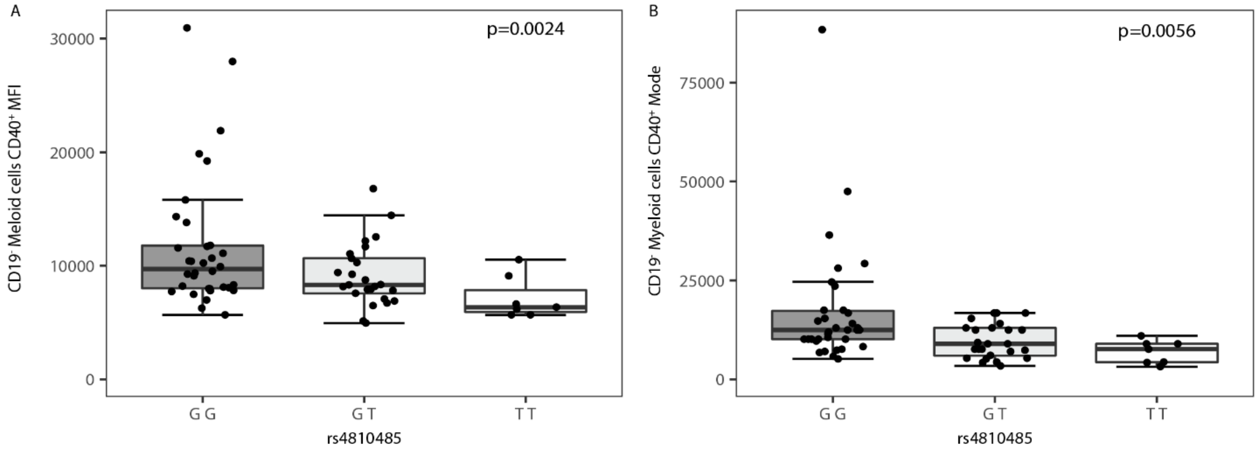

Supplementary Figure 11. Association of the *CD86* SNP rs9282641 with B cell surface expression of CD86 in healthy controls. Mean fluorescent intensity ratio of CD86 by genotype is depicted **A.** B cells, **B.** naïve B cells **C**. non-switched memory B cells and **D.** class-switched memory B cells. Box-whisker plots represent median, quartiles and 1.5x interquartile range. rs9282641*G is the multiple sclerosis risk allele.

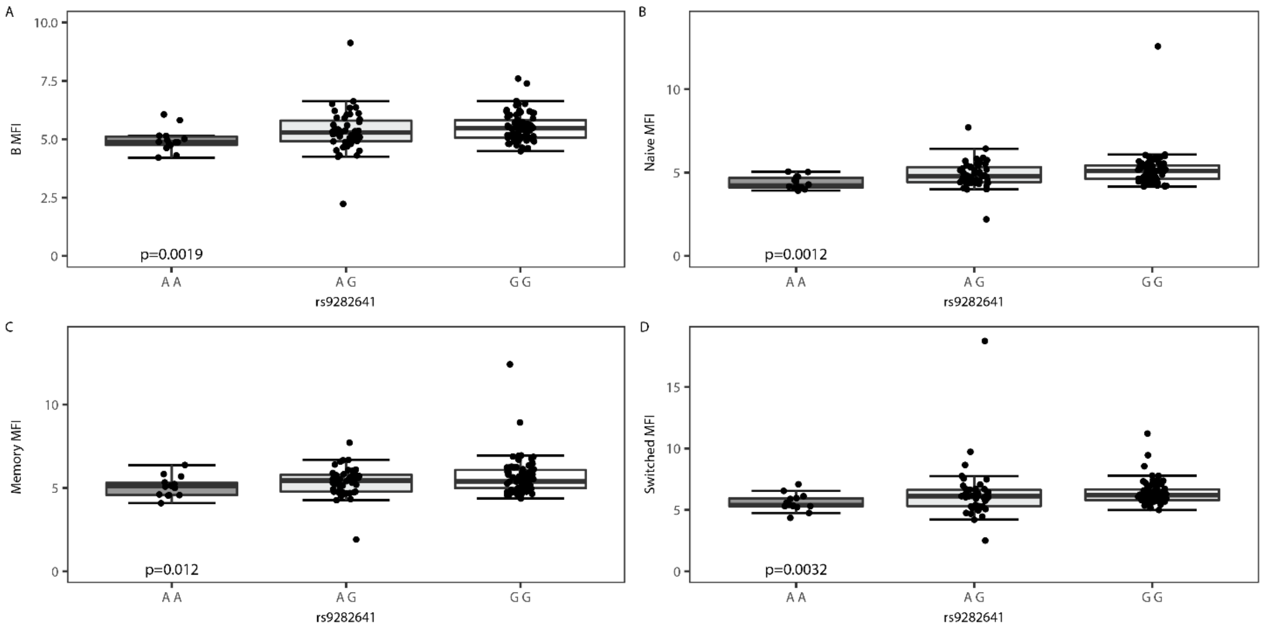

Supplementary Figure 12. Association of the *CD86* SNP rs9282641 with B cell surface expression of CD86 in untreated multiple sclerosis patients. Mean fluorescent intensity of CD86 by genotype is depicted for **A.** B cells, **B.** transitional B cells, **C.** naïve B cells, **D.** non-switched memory B cells, **E.** class-switched memory B cells and **F.** plasmablasts. Box-whisker plots represent median, quartiles and 1.5x interquartile range. rs9282641*G is the multiple sclerosis risk allele.

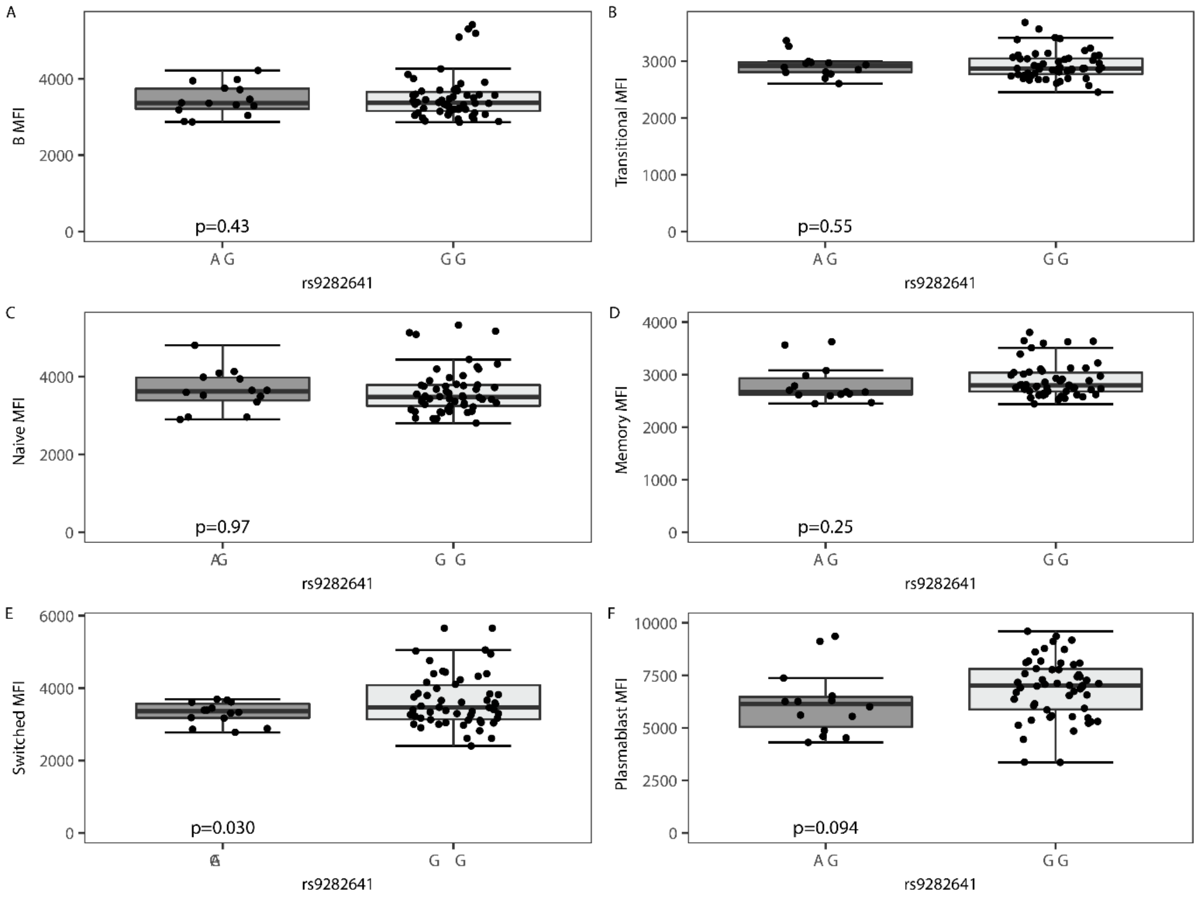

Supplementary Figure 13. Association of the *CD86* SNP rs9282641 with monocyte surface expression of CD86 in healthy controls. Percentage of CD86 positive cells by genotype is depicted for **A.** total, **B.** classical, **C.** intermediate and **D.** non-classical monocytes. Mean fluorescent intensity ratio of CD86 by genotype is depicted for **E.** total, **F.** classical, **G.** intermediate and **H.** non-classical monocytes. Box-whisker plots represent median, quartiles and 1.5x interquartile range. rs9282641*G is the multiple sclerosis risk allele.

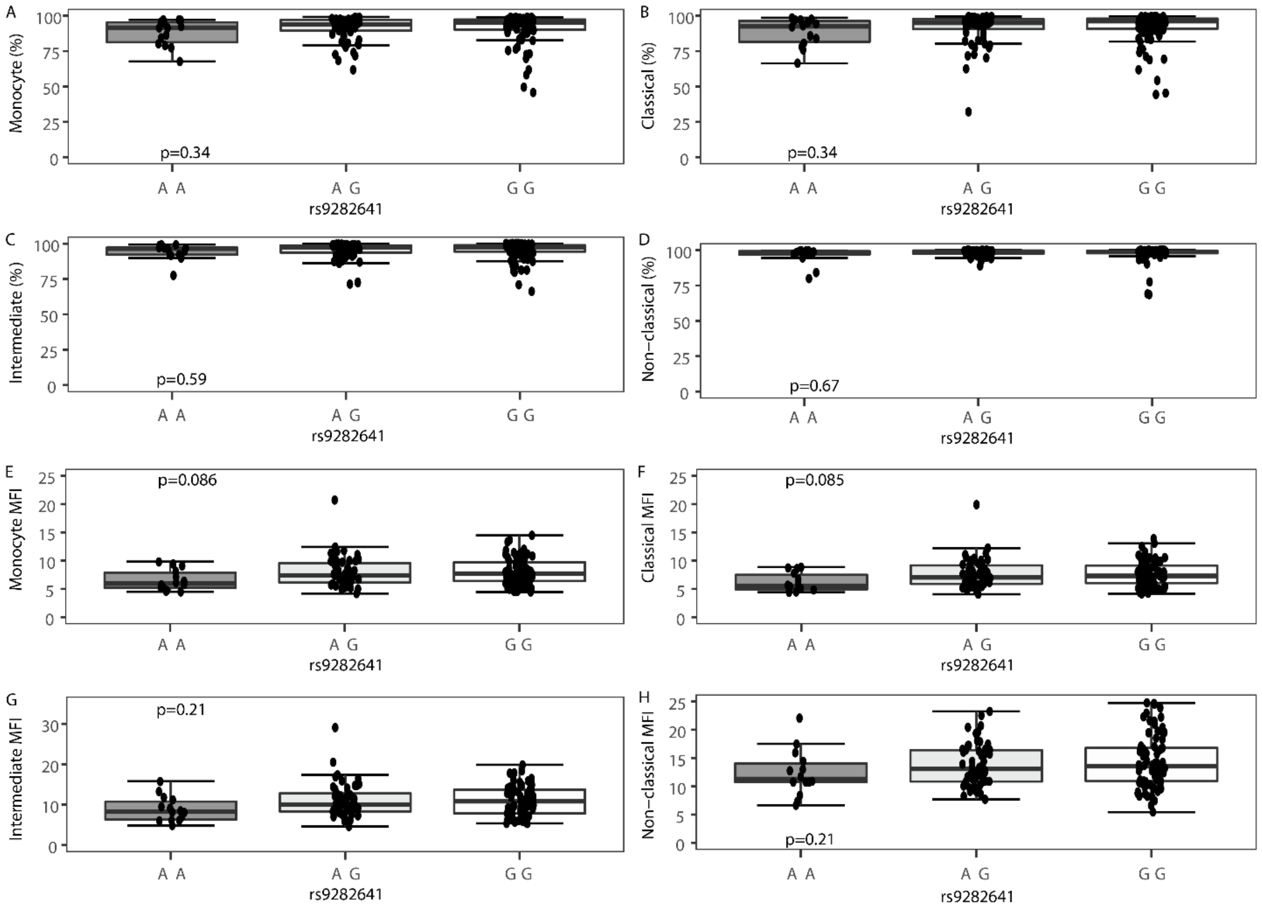

Supplementary Figure 14. Association of the *CD86* SNP rs9282641 with surface expression of CD86 in the myeloid subset of untreated multiple sclerosis patients. A. Mean and **B.** mode of fluorescent intensity of CD86 by genotype is depicted in $CD86^{+}$ $CD19^{-}$ myeloid cells. Box-whisker plots represent median, quartiles and 1.5x interquartile range. rs9282641*G is the multiple sclerosis risk allele.

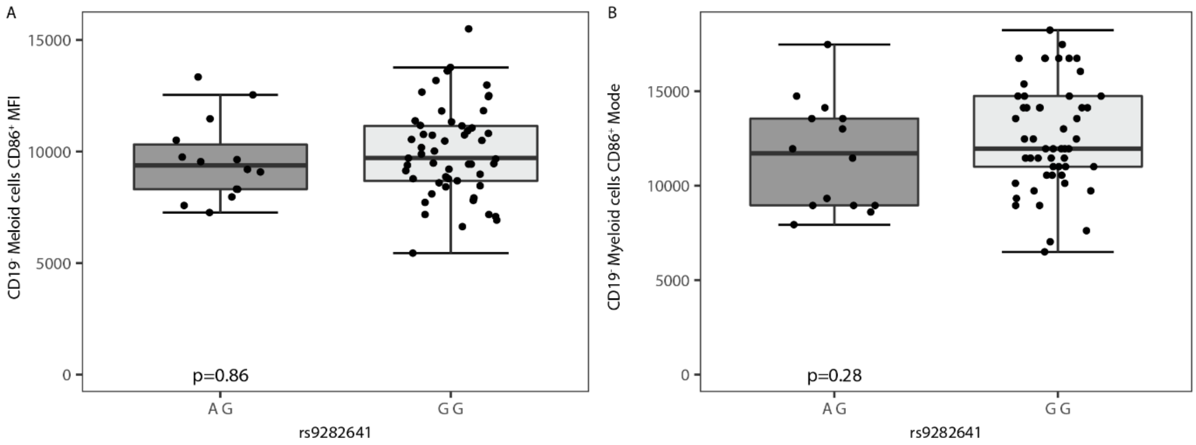

Supplementary Figure 15. Genotype specific expression of CD86 alternative splice forms in B cells of healthy controls. Relative quantity (RQ) of CD86 transcripts in B cells **A.** containing the transmembrane domain, **B.** lacking the transmembrane domain, **C.** starting with the first exon and **D.** starting with the second exon. Box-whisker plots represent median, quartiles and 1.5x interquartile range. rs9282641*G is the multiple sclerosis risk allele.

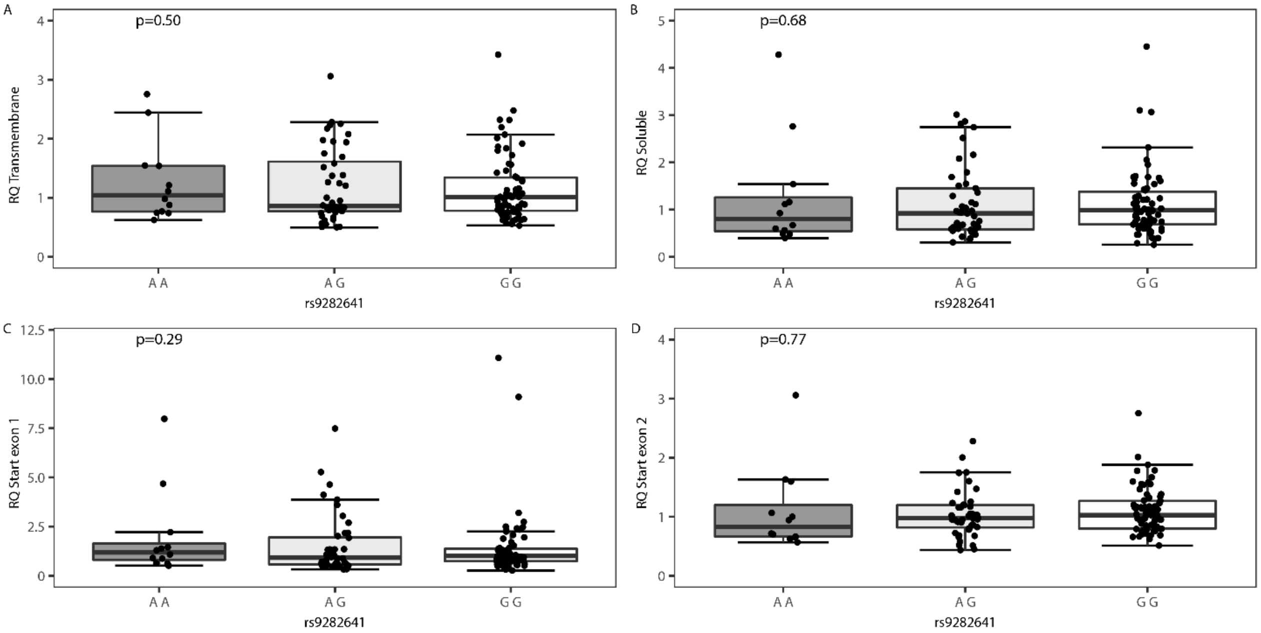

Supplementary Figure 16. Genotype specific expression of CD86 alternative splice forms in monocytes of healthy controls. Relative quantity (RQ) of CD86 transcripts in monocytes **A.** containing the transmembrane domain, **B.** lacking the transmembrane domain, **C.** starting with the first exon and **D.** starting with the second exon. Box-whisker plots represent median, quartiles and 1.5x interquartile range. rs9282641*G is the multiple sclerosis risk allele.

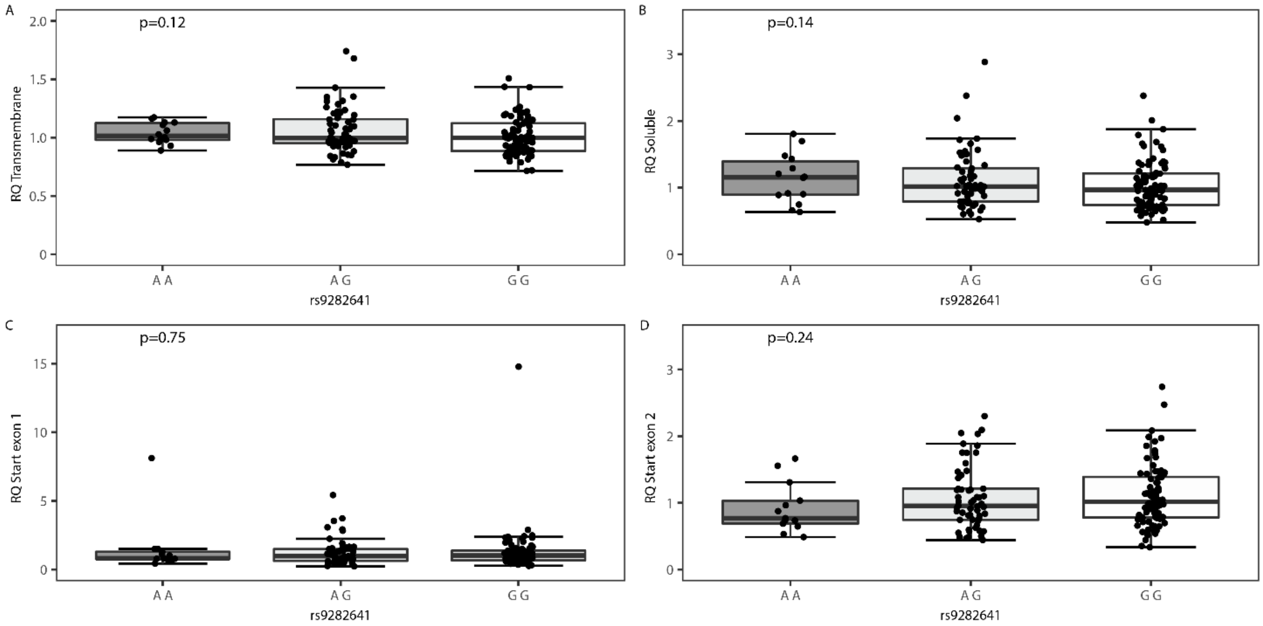

Supplementary Figure 17. Genotype specific expression of soluble CD86 in serum of healthy controls. rs9282641*G is the multiple sclerosis risk allele.

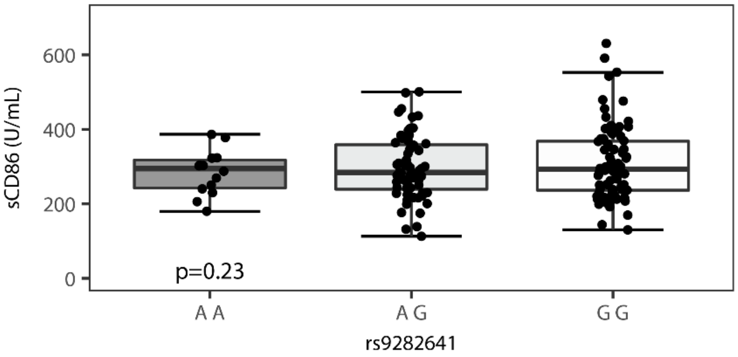

Supplementary Figure 18. Longitudinal IL-10 measurements in untreated multiple sclerosis patients (N=17). A. Correlation between IL-10 measurements in samples obtained at an average three-year interval, in 2014 and 2017, both performed with the V-Plex human Proinflammatory panel Meso Scale Discovery plate. **B.** Association of the CD40 SNP rs4810485 with serum IL-10 levels in the smaller 2014 dataset. The direction of the genotype - IL-10 effect is the same as in the total 2017 dataset, although the power is substantially more limited as this 2014 subset represents 25% of the total 2017 dataset in Figure 6 (N = 66). Box-whisker plots represent median, quartiles and 1.5x interquartile range. rs4810485*T is the multiple sclerosis risk allele.

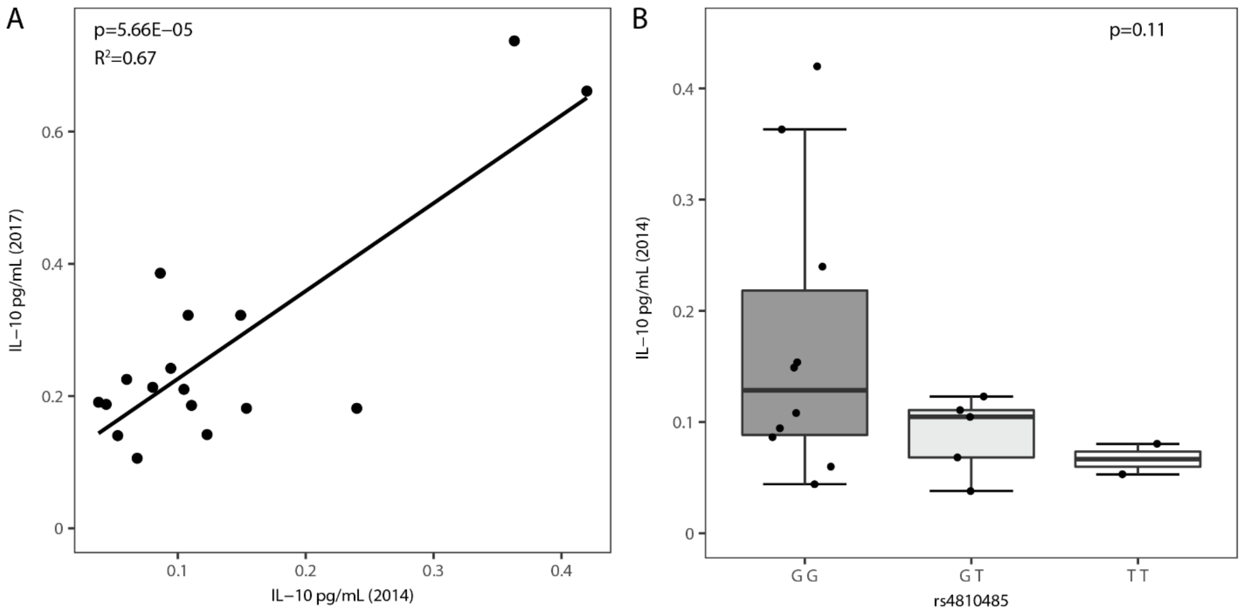

Supplement: Supplementary Data [file awx372_brain-2017-00934-file008.pdf]
